# Access to online learning: Machine learning analysis from a social justice perspective

**DOI:** 10.1007/s10639-022-11280-5

**Published:** 2022-10-04

**Authors:** Nora A. McIntyre

**Affiliations:** Southampton Education School, University of Southampton, Building 32, University Rd, Highfield, Southampton, SO17 1BJ UK

**Keywords:** Machine learning, Online learning, COVID-19, Country inequalities, Educational access

## Abstract

Access to education is the first step to benefiting from it. Although cumulative online learning experience is linked academic learning gains, between-country inequalities mean that large populations are prevented from accumulating such experience. Low-and-middle-income countries are affected by disadvantages in infrastructure such as internet access and uncontextualised learning content, and parents who are less available and less well-resourced than in high-income countries. COVID-19 has exacerbated the global inequalities, with girls affected more than boys in these regions. Therefore, the present research mined online learning data to identify features that are important for access to online learning. Data mining of 54,842,787 initial (random subsample n = 5000) data points from one online learning platform was conducted by partnering theory with data in model development. Following examination of a theory-led machine learning model, a data-led approach was taken to reach a final model. The final model was used to derive Shapley values for feature importance. As expected, country differences, gender, and COVID-19 were important features in access to online learning. The data-led model development resulted in additional insights not examined in the initial, theory-led model: namely, the importance of Math ability, year of birth, session difficulty level, month of birth, and time taken to complete a session.

## Introduction

Online learning has long been hailed as an avenue for increased access to education (Carlsen et al., 2016), with many holding huge hopes of online learning as an engine for social change (Geith & Vignare, 2008). When online learning is designed in an inclusive manner (Adam, 2020a), online learning can be as effective as in-person education if not more, depending on learner preferences (Smith et al., 2000). Furthermore, online learning has been viewed as an important avenue for supplementing where in-school provision is lacking: these include offering courses not available at school; targeting learners who are less well supported in school; addressing timetabling conflicts in school; and offering advanced learning (Butler Kaler, 2012; Picciano et al., 2010). Indeed, there is evidence that online learners are typically those who cannot afford additional in-person learning provisions to supplement school provisions (Moloney & Oakley, 2010). A recent meta-analysis supports the potential for online learning to improve learning outcomes in low-and-middle-income countries (LMICs; Major & Francis, 2020). Thus, the impression has been that online learning would best serve the under-served, due to the flexibility that online learning offers, especially those living in remote locations and for whom complex home lives (Bakia et al., 2012), including out-of-school (Colwell et al., 2018), at-risk (Lewis et al., 2014), and girl (Jiang et al., 2018) children.

Yet, access to online learning is itself not an easy feat. Learners need to have high degrees of independence, self-regulation, and motivation in order to maintain their own access to online learning (Cho & Shen, 2013; Kim & Frick, 2011). Interpersonal presence is known to be supremely important for effective learning (Pianta et al., 2014) but, in online learning, manifests very differently from how it does classroom learning ─ and does so in complex ways for adequate teacher presence (Avery, 2018) and for peers presence (Akcaoglu & Lee, 2016). Moreover, stark between-country differences exist in the extent to which learners can make use of technologically demanding educational resources such as online learning, and limiting potential to access such resources. The disadvantage particularly applies to LMICs, such as Kenya and Thailand (OECD, 2019). In such regions, home connectivity is even more strained than in schools, making access to online learning nearly impossible (Aboagye et al., 2021). Yet, it is learners in such homes that online learning is most needed, as these are where parents are much less likely to be available or adequately resourced than those in high-income countries (Khlaif et al., 2021). Furthermore, the female disadvantage in accessing classroom learning is typical in classroom learning across LMICs (Jafree, 2021): the same constraints can apply to online learning in the home if not more since, compared with boys, girl learners are having to learn in the home context where home responsibilities are more salient than in school (Jones et al., 2021). The Pandemic has exacerbated all these challenges, widening digital divides more than ever. Indeed, the Pandemic exacerbated inequalities as the poor found themselves unable to compensate for lost school resources (Al-Salman & Haider, 2021), whilst the girls’ home responsibilities exponentiated (Mathrani et al., 2021).

### Significance of the present study and research questions

Online learning is typically regarded as an equaliser that grants educational access to the under-served. Yet, the opposite is often true among the most marginalised, with disparities exacerbated rather than alleviated in online learning, due to digital divides that are not present in classroom learning (Mathrani et al., 2021). Previous research has considered access to online learning primarily either through small-scale studies involving stakeholder reported experiences (Biswas et al., 2020) and ability testing (Coiro, 2011), or through large-scale econometric analyses of national census data (Madaio et al., 2016) and literature syntheses (Reinders et al., 2021). Known research has yet to harness online learning data itself to understand predictors (or ‘features’) of access to online learning, let alone to capitalise on the potential of analytic capabilities offered by data mining with machine learning techniques. Where similar analyses have been conducted on online learning, these have focused on affluent populations and on learning outcomes (Hung & Crooks, 2009; Peach et al., 2021), rather than on equity-related features and outcomes. Previous machine learning analysis of the online learning data has demonstrated that one of the most important features in predicting the online learning outcomes is cumulative experience (McIntyre, under review). But what predicts cumulative experience: that is, what are the most important features in accessing online learning?

The present article reports research that advances the existing body of research on access to online learning. To do this, machine learning analysis was deployed on online learning data to understand features relating to social justice that predict access to online learning. By investigating country, gender, and COVID-19, it was hoped that features relating to digital divides would be understood in terms of their importance in an outcome that is foundational to students’ opportunity to engage in education: that is, their access to online learning. Moreover, this research implements machine learning analysis that partners theory with data to derive analytic insights, so as to maximise the potential lessons both from domain expertise and from data-led model optimisation (Chen et al., 2020; Rosé et al., 2019). Thus, the analytic process began with the following domain-led hypotheses:

1. Given country differences in the infrastructural and literacy constraints for accessing online learning, the country setting will be found to have importance in predicting access to online learning outcomes.
2. Given gender differences in the sociocultural and biological challenges to educational access, gender was expected to have importance in predicting access to online learning.
3. The online learning necessitated by the emergency school closures during COVID-19 were expected to have importance in students’ access to online learning.
  Moreover, insights were anticipated to emerge from data-led model optimisation. As such, the fourth hypothesis was as follows:
4. Data-led feature selection will identify features unanticipated by the present theoretical framework that have importance in predicting access to online learning.

## Theoretical framework

### Country differences in access to online learning

#### Infrastructural barriers

Access issues in online learning can be largely attributed to infrastructural challenges. Although analog technologies such as radio (Damani, 2020) and television (Watson & McIntyre, 2020) have been recognised for their benefits to widening educational access, internet connectivity is essential to the use of most digital technologies and is thus a source of significant disparities. LMICs face a dramatic disadvantage in their access to connectivity in contrast to high-income countries, with 5% with home broadband subscription in Sub-Saharan Africa (South Africa) and Southeast Asia (Malaysia) versus > 40% with home broadband subscription in Great Britain (OECD, 2019). A corresponding LMIC-related difference is observed regarding the broadband download speeds. High-income countries (HICs) such as Great Britain have download speeds of approximately 60Mbps, whereas LMICs suffer from much slower download speeds: just under 20Mbps in Southeast Asia, and even lower in Sub-Saharan Africa (< 10Mbps; OECD, 2019). Other than prohibitive pricing for securing any bandwidth at all, others make do with the bandwidth they have by downloading learning materials: however, the bandwidth speed is so slow that learners often do not have time to download everything they need in order to progress their learning. So, the bandwidth poverty cascades into a poverty of time (Madaio et al., 2016).

Correspondingly, between-LMIC differences have been observed in access to electricity access, with Southeast Asian countries attaining > 90% and Sub-Saharan Africa still at 45% by 2018 (Shyu, 2022): this further reflects the between-LMIC differences in the priority given by gatekeepers to digital access for all, as well as the Sub-Saharan African reluctance to diversify beyond rural economies (van Donge et al., 2012). Beyond the net availability of electricity at national level, the reliability of electricity provision is another problem among the most deprived within LMICs, who often live where electricity shortages are a norm (Dhawan, 2020). Indeed, electricity is an important predictor of internet access (Houngbonon & Le Quentrec, 2020) which in turn predicts online learning. Therefore, electricity stands as an obstacle in access to online learning.

#### Literacy barriers

Other than infrastructure being crucial for access to online learning, learners’ literacy is important to enable access to the content of online learning. Literacy in English seems most relevant, as this is the language used in most online learning content, as languages native to online learners in LMICs are not established or prominent in contrast to English (Osborn, 2006). The role of English literacy is second only to income in predicting internet access in Sub-Saharan Africa (Houngbonon & Le Quentrec, 2020). This is echoed in other LMICs, where students’ interest in (Lamb & Arisandy, 2020) and progress with (Meurant, 2010) developing English as an ‘additional language’ correlates with use of online learning. In fact, Southeast Asian regions such as Thailand are documented to have exceptionally low English language proficiency, especially when compared with Kenya which has high proficiency in English (Education First, 2021; Yang, 2004),^1^ perhaps for cultural reasons (Young, 2021). If English literacy is more important than infrastructure in predicting access to online learning, then Thailand may be found to have lower access even than Kenya. Thus, literacy, especially in English, can itself bring about between-country disparities in access to online learning.

Other than English literacy, digital literacy is crucial too. Learners in LMICs may at times have high English literacy levels, but low digital literacy due to infrastructural constraints in this area of development which, in turn, limits learners’ access to online learning content that is otherwise widely available (Daniel et al., 2015). Once inside an online learning environment, digital literacy is then relevant for navigating the environment in order to engage with and utilise it as a resource (Askov et al., 2003). Moreover, online learners in LMICs have been documented not to be able to transfer digital skills from technologies in educational settings to those in personal settings, such as when they stop using school computers to use their home computers or laptops (Winke & Goertler, 2008). So, digital literacy is critical for learners’ access to online learning content (Queiros & Villiers, 2016). Indeed, online reading comprehension is a capability in its own right, as distinct from offline (i.e., paper-based) reading capability. Such digital literacy is so important in online learning that it compensates for a lack of prior subject knowledge to enable learning outcomes on a par with learners with high prior subject knowledge (Coiro, 2011).

### Gender differences in access to online learning

Learners in LMICs face access challenges that learners in HICs never need to contend with, but gender differences exist (Reinders et al., 2021; Whetten et al., 2011). Boys in LMICs face more hindrances to growth as well as harsher discipline than girls (Bornstein et al., 2016). Boys are also more likely to be involved with work outside the home, or family business, than girls in LMICs (Putnick & Bornstein, 2016). However, girls are more likely to be involved with excessive household chores than boys (Putnick & Bornstein, 2016, cf. Whetten et al., 2011). Thus, whilst both girls and boys in LMICs face more challenges and responsibilities than those in HICs, it is the girls who face the domestic challenges and, therefore, the most involved and all-encompassing obstacles to learning.

Cultural obstacles exist that prohibit girls from learning more than boys. In Southeast Asian homes, girls are advised against dominating in the classroom and in academic achievements: this is so as to maintain cultural expectations and ensure the girls can ultimately secure a husband (Conchas, 2006; Pataray-Ching et al., 2006; Robbins, 2004). At times, the cultural expectations are transmitted in the manner of “hidden curricula” in LMICs (Mollaeva, 2018). Related, household chores and family care responsibilities typically get allocated to girls (Armstrong-Carter et al., 2020) who are additionally rewarded for early motherhood (Donnelly, 1991), whilst academic development is allocated to boys more than to girls (Goldstein, 1985). A similar sociocultural framework is found in Sub-Saharan Africa, with girls more likely than boys to perform household chores during the week and during school hours (Agesa & Agesa, 2019; Tian, 2019) and across LMICs more generally (Putnick & Bornstein, 2016). Accordingly, chores have been found to affect girls’ learning outcomes more than boys in Sub-Saharan Africa (Tan et al., 2022). The chores further related to greater prevalence of stress-related challenges among girl learners in comparison with boys (Beattie et al., 2019).

Furthermore, there are biological obstacles. Gender-related disadvantages can stem from monthly cycles faced exclusively by girls, regardless of country income level. Such ‘period poverty’ is especially a challenge for girls in LMICs who are less likely to have relevant, sanitary napkins to hand, which can prevent them from getting on with learning (Bakibinga & Rukuba-Ngaiza, 2021). Among Sub-Saharan African girls, their culturally established responsibility of water-fetching only compounds gender inequalities as the water is often not sanitary, subjecting girls to longer term ill-health which further prevents girls from learning (Miiro et al., 2018; Sommer et al., 2017).

Corresponding gender differences have been found in online learning. Girls use the internet less than boys, and go onto develop digital skills less completely than boys do, regardless of country income level (Kashyap et al., 2020). The gender disparity in internet access is worse in LMICs, with girls using the internet significantly and consistently less than boys, whether learning occurs in urban, rural, or remote island settings (Sujarwoto & Tampubolon, 2016). Correspondingly, girls have been found to use online materials and to engage in any kind of learning significantly less than boys in LMICs home (Jones et al., 2021).

### The role of COVID-19 in access to online learning

Online learning during the Pandemic’s emergency school closures has been applauded for increasing learner agency by removing the need to travel and increasing learner flexibility, both in LMICs (Biswas et al., 2020; Mathrani et al., 2021) and in HICs (Laufer et al., 2021). In fact, COVID-19 has been found to improve Mathematics development through online learning (McIntyre, under review^2^).

Nevertheless, the Pandemic has been reported to bring much damage on the whole, setting back the most deprived within (Agostinelli et al., 2022; González & Bonal, 2021; Nevická & Mesarčík, 2022) and between (Laufer et al., 2021) countries dramatically, as those in homes that could afford to respond with resource compensation raced ahead with continued access to academic learning, leaving other learners behind (Ferri et al., 2020). Moreover, even in HICs, non-learning demands spiked during emergency school closures which impacted upon learners’ capacity to engage with online learning, whilst lower levels of pre-Pandemic digital competencies stood as an obstacle (Hews et al., 2022; Mok et al., 2021). All this was in addition to the nature of the home environment which is generally less conducive to learning than school settings, regardless of the country income level (Yates et al., 2021).

Furthermore, COVID-19 exacerbated pre-existing inequalities, including country disparities especially in terms of infrastructure. Learners in LMICs typically named internet connectivity to have been the primary challenge of online learning during COVID-19 (Aboagye et al., 2021; Khlaif et al., 2021). Learners in LMIC homes were even less able to compensate for lost access to learning resources than those in deprived parts of HICs (Al-Salman & Haider, 2021; Khlaif et al., 2021), making the loss of teacher support and school resources more damaging in LMICs than in HICs. COVID-19 exacerbated, too, the gender disparities in access to online learning, with girls more likely to report home-related obstacles to online learning than boys during the Pandemic (Jafree, 2021; Mathrani et al., 2021), especially among learners who had been accustomed to in-school learning as opposed to out-of-school (or informal) learning (Reich et al., 2013; Tan et al., 2022).

### Machine learning for online learning analysis

The growing prevalence of online learning (OECD, 2017) was catalysed by COVID-19 and is now an established reality (OECD, 2020). Online learning has thus become a normative context from which to understand learning patterns.

Online learning research brings with it challenges of big data, which need to be met with analytic tools appropriate to big data, in order to address the unsuitability of traditional statistical techniques for the high dimensionality of big data (Fan et al., 2014). The use of appropriate analytic tools involves a paradigm shift within the analyst which, beyond a shift in language (Hassibi, 2016), to a shift in culture and goals (Friedman, 1998). That is, to shift from reliance upon stochastic modelling for understanding and interpreting mechanisms, to the use of algorithmic model development for an explanation that incorporates complexity in the real-world (Breiman, 2001). So, in inferential statistics, analytic models are viewed as a final theoretical framework to be developed a priori then verified using data, such that model optimisation for maximum model fit is viewed as over-saturation or analytic ‘cheating’. In contrast, machine learning aims to optimise analytic models through theory, algorithms, and data via multiple iterative cycles, before finally scrutinising the best-fitting model for insights into a problem and real-world decisions (Orrù et al., 2020). Thus, there is a fundamental shift in the way modelling is viewed and used in machine learning as compared with traditional, inferential statistics.

Other than the shift in mindset with regard to analytic models, the role of human expertise is being increasingly emphasised with regard to machine learning model development: hence the term, *human-in-the-loop* (Cranor, 2008; Dautenhahn, 1998; Grønsund & Aanestad, 2020). The central importance of humanity in the use of machine learning for educational research and practice has been unpacked very recently by Khosravi and colleagues (Khosravi et al., 2022) in their framework of explainable artificial intelligence for education (XAI-ED). Within this framework, six priorities are proposed, including centrality of stakeholders (e.g., learners, parents, teachers), avoidance of common pitfalls in the use of machine learning (e.g., overly complex models), and thoughtful explanations (i.e., effective and relevant demonstrations and examples). In all, two implications arise from the importance of human involvement that are implemented in the present analyses.

Firstly, when humans are the end-users of the analytic outcomes, then analytic models must finally be interpretable by humans: indeed, humans are almost always end-users of machine learning models at some level, whether as experts, lay-users, or ethically involved (e.g., with potential to bear the consequences of ‘prejudiced’ decision-making based on unrepresentative data; Doshi-Velez & Kim, 2017). With the complexity of machine learning algorithms, interpretability does not come directly from the algorithm itself, but instead through an explanation model. For this, Shapley values have been proposed (Lundberg & Lee, 2017) which are ‘model agnostic’: that is, applicable to machine learning models, regardless of the algorithm used (Watson, 2021). Shapley values are fully ‘additive’ too, meaning that they possess all the properties relevant to additive feature attribution, and so completely “attribute[s] an effect to each feature and, [by] summing the effects of all feature attributions, approximates the output of the original [analytic] model” (Lundberg & Lee, 2017, p. 2).

Secondly, because humans are the end-users of analytic outcomes, humans should be involved in the development of the analytic model (Zhou et al., 2017). Ultimately, interpretability and usefulness of a machine learning model is domain specific (Carvalho et al., 2019). Therefore, model development should involve humans, especially for domain expertise which is unrivalled by algorithms and data in terms of relevance, complexity, and comprehensiveness. In fact, when domain expertise is involved, the predictive performance of final models exceeds the performance of models developed using data and algorithms only (Roccetti et al., 2020).

## Method

**Fig. 1:**
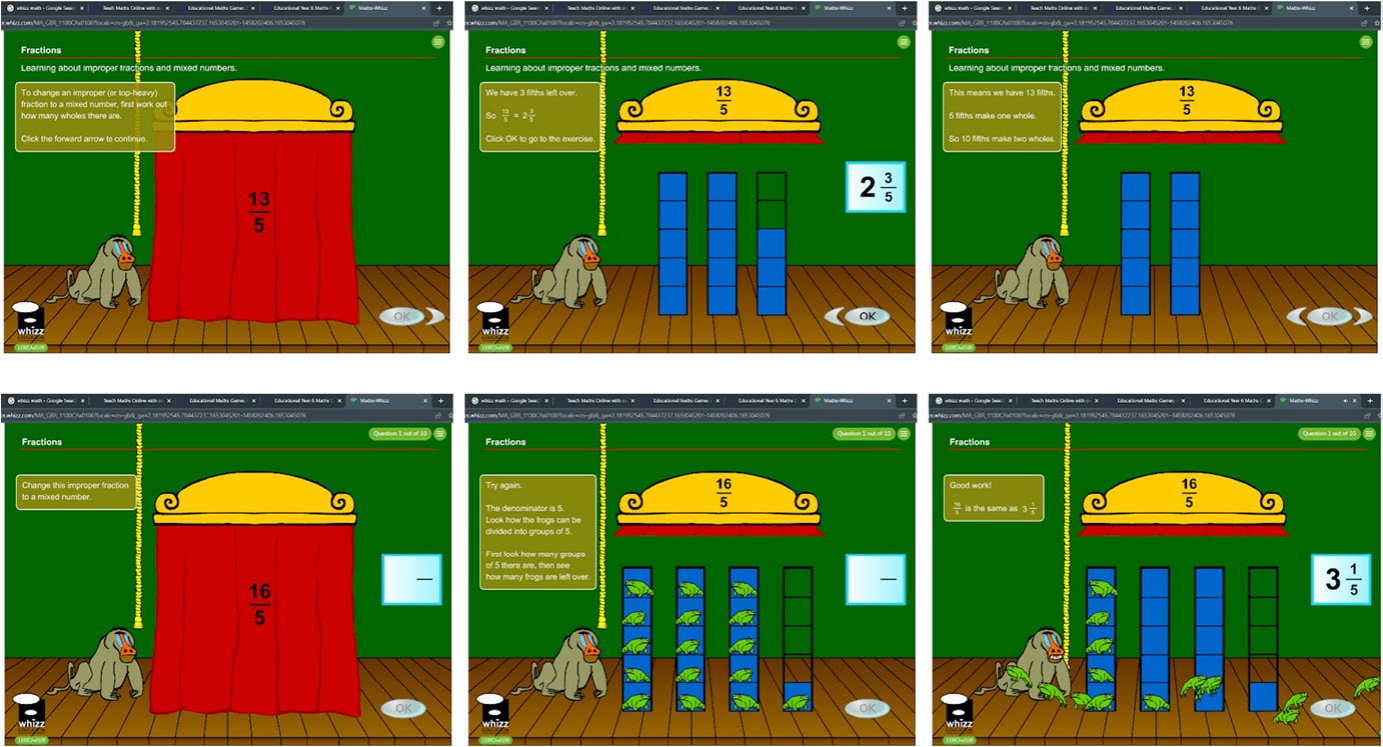
Screenshots from Year 6 Maths Games demo. Screenshots progress from left to right, first the top row, then the bottom row. (See https://www.whizz.com/maths-games/year-6-maths-games.)

Secondary data analysis was conducted on data from an online intelligent tutoring system called the Maths-Whizz Tutor by Whizz Education. In accordance with the potential for social good that online learning offers, the Maths-Whizz Tutor targets deprived regions in partner countries. Whizz is co-developed with local stakeholders, so data from this specific platform carries exceptional ecological validity and contextual sensitivity. Meanwhile, The scaled nature of this pre-existing means more data is available for analysis—and for this data to derive from a platform genuinely used and valued by learners in the original contexts. Moreover, the curriculum is developed in partnership with local stakeholders to ensure that the content is contextually relevant (Adam, 2020b). Example screenshots from the online learning environment are shown in Fig. 1.

The Maths-Whizz Tutor covers 22 age-appropriate topic areas in Mathematics, which break down into 1222 learning objectives (i.e., lessons). Log files shared from this platform spanned the years 2016 to 2020 inclusive. Each data point from this platform represented one completed lesson, which involved an exercise and a test. For each lesson datapoint, an anonymised pupil ID was provided, and each pupil was linked with an anonymised school ID. Only completed exercises are included for analysis. Although the laptop was data science ready (see Apparatus for details), a random subsample of n = 5000 (seed = 1) still needed to be taken from the whole sample of n = 54,842,787, in order to enable the computational processing demands of the large dataset and advanced analyses implemented in this study.

### Participants

Whizz Education by Math Whizz is designed for and accessed by learners aged between 5 and 13 years. The platform is available for use in schools and in the home: during the COVID-19 pandemic, data was collected solely from the home context for a significant period, whereas it was collected from both schools and homes before onset of the pandemic. Within the random subsample of n = 5000, n = 2581 were male and n = 2418 were female.

The platform is available to multiple countries, three of which were sampled in the present analyses: namely, Kenya, Thailand, and the UK. Thus, two low-and-middle-income countries (LMIC; i.e., Kenya and Thailand) and one high-income country (the UK) were sampled.^3^ This enabled between-culture (Kenya vs. Thailand vs. the UK) and between-income-status (Kenya and Thailand vs. the UK) comparisons. Within the random subsample of n = 5000, n = 1755 (n_Male_ = 1012) data points were from Kenya, n = 1128 (n_Male_ = 479) were from Thailand, and n = 2117 (n_Male_ = 1090) were from the UK.

The data analysed during the current study are not publicly available due to data ownership by Math Whizz but availability can be discussed with the corresponding author upon reasonable request.

### Apparatus

Computationally powerful laptops were used for this analysis: MSI Stealth, NVIDIA RTX 3060 GPU, 16 GB RAM, 2.60 GHz, 500 GB SSD (laptop 1); Dell Precision 7560, NVIDIA RTX A5000, 32 GB RAM, 4.80 GHz, 1 TB SSD (laptop 2). The two devices were comparable in computational power and performance. Although the laptop was data science ready, a random subsample of n = 5000 (seed = 1) still needed to be taken from the whole sample of n = 54,842,787, in order to enable the computational processing demands of the large dataset and advanced analyses implemented in this study. Note that the wisdom in random sub-sampling for data mining is recognised in the field (Attewell & Monaghan, 2015; Bouckaert & Frank, 2004; King & Resick, 2014; Ratner, 2011; Sculley & Pasanek, 2008).

Jupyter Notebook and locally hosted Google Colab were used. The Python libraries used for the present analyses include Vaex for basic manipulation of hdf5 files (Breddels & Veljanoski, 2018) in parallel with Numpy (Harris et al., 2020), Pandas for major data manipulation (McKinney, 2010). Sklearn (Pedregosa et al., 2011) was used to run linear regression, elastic net cross-validation, regression with elastic net penalty, lasso cross-validation, regression with lasso penalty, and to convert data to DMatrix for XGBoost (T. Chen & Guestrin, 2016). Shapley values and related visualisations were obtained through shap.Explainer method from the SHAP library (Lundberg & Lee, 2017).

### Measures

The outcome variable was *play_count* which was the total number of lessons that the learner had complete, including the one being completed at the time of data collection.

#### Features

All the features available for selection in this analysis are listed in Table 1: all of these were initially included in Phase 2 for data-led feature selection and model development, whereas only *country, gender,* and *since_covid* were included in Phase 1 for the theory-led feature selection.

**Table 1:** Feature dictionary, with all features initially included in analysis (i.e., Phase 2 model development)

	Feature name	Feature explanation	Feature engineering process
1	*topicId*	Topic identifier (22 topics in total)	None; from log file
2	*mathLevel*	Academic difficulty of the *lesson* (not level of the child). The academic difficulty was framed in terms of the academic age targeted by a lesson and was divided by quarters of a year (i.e., one year divided into 0.25, 0.50, 0.75, and 1.00)	None; from log file
3	*exerciseId*	Within each quarter, exercises were sequenced in order of difficulty and ranged from 100 to 1000 (i.e., 100, 200, 300, etc.), incrementing at intervals of 100 within each quarter then resetting at the next quarter	None; from log file
4	*stackDepth*	The feature, stackDepth, related to the lesson’s mode, with the default value being stackDepth = 1 to signify progression; if a learner failed a default, progression lesson, they would regress to a simpler exercise in to a lesson mode with stackDepth = 2; failing that, the learner would be regressed further to even simpler exercise at stackDepth = 3. If the learner passed the stackDepth = 3 exercise and test, they would move back to complete the exercise and test at stackDepth = 2 then, if they pass that test, return to the lesson at stackDepth = 1	None; from log file
5	*timeTaken*	How long the learner took to progress from beginning of lesson to the end, including the exercises and test	None; from log file
6	*questionTime*	How long the learner took to complete the exercise questions	None; from log file
7	*tutorialTime*	How long the learner took to complete the tutorial as a whole	None; from log file
8	*totalQuestions*	The number of questions that the learner attempted in that lesson	None; from log file
9	*lesson_type*	The default progression tutor exercise, regression tutor exercise, replay exercise, tutor test	None; from log file
10	*total_help*	The number of times help was sought by the learner	None; from log file
11	*replay*	A summary feature indicating whether the lesson was a standard, progression one, or whether the learner was repeating the lesson for whatever reason	None; from log file
12	*markedYear*	*2016, 2017, 2018, *etc	Computed from log file variable, *marked* (e.g., 30/01/2020 07:40)
13	*markedMonth*	*January* = *1, February* = *2, *etc	Computed from log file variable, *marked* (e.g., 30/01/2020 07:40)
14	*markedWeek*	*1 to 52 for each calendar year*	Computed from log file variable, *marked* (e.g., 30/01/2020 07:40)
15	*since_covid*	*1* = *2020; 0* = *2016 to 2019*	Computed from log file variable, *marked* (e.g., 30/01/2020 07:40)
16	*Male*	Dummy variable (or one-hot coding)	Dummy generated from *gender* (original variable)
17	*Female*	Dummy variable (or one-hot coding)	Dummy generated from *gender* (original variable)
18	*play_count*	The total number of lessons completed by each learner	Computed from log file variable, *anonymised_pupil_id* (e.g., 88,873,931)
19	*birthYear*	Year of birth	Computed from log file variable, *date_of_birth* (e.g., 01/01/2006)
20	*birthMonth*	Month of birth	Computed from log file variable, *date_of_birth* (e.g., 01/01/2006)
21	*pupil_ageQuart*	The learner’s age in quarters. That is, year + quarter, e.g., 12.25 for 12 years and a quarter; births between January and March were quarter = 0, births between April and June were quarter = 0.25, etc	Computed from log file variable, *date_of_birth* (e.g., 01/01/2006)
22	*mathAbility*	Learner academic age. For example, a learner with pupil_ageQuart = 12.25 years who is attempting a lesson with mathLevel = 9.25 will be showing the mathAbility of + 3 years	Computation: *pupil_ageQuart*—*mathLevel*
23	*Kenya*	Dummy variable (or one-hot coding). 1 = Kenya, 0 = UK or Thailand	Computed using *Kenya* data file as reference
24	*UK*	Dummy variable (or one-hot coding). 1 = UK, 0 = Kenya or Thailand	Computed using *UK* data file as reference
25	*LMIC*	Dummy variable (or one-hot coding). 1 = LMIC (Kenya or Thailand), 0 = HIC (UK)	Computed from *Kenya* and *Thailand*
26	*InCountryDep*	1 (least deprived) to 3 (most deprived) using country-specific deprivation codes as applied at school level. Missing data were replaced by the sample-level mean (i.e., 2.08) and rounded to the nearest integer (i.e., 2) Additional notes: The UK deprivation status was calculated using the Index of Multiple Deprivation 2019 (IMD2019, Penney, 2019). This is a decile index which was split into three bins using the Pandas cut function. The Kenyan deprivation status came as a three-level feature, with urban being the most well-resourced, rural as middling, and hardship as the least well-resourced learners. The Thai deprivation stati came as a three-level feature: private or independent schools were rated to be the most well-resourced, followed by provincial public schools, and rural public schools as the least well-resourced	Computed from log file variable, *deprivation*

More specifically, In Phase 1, the analytic model was theory-led and based on the author’s domain expertise, the established literature for identification of the most important constructs, and the most robust measures in my data as representatives of the most relevant constructs identified from initial data analysis when theoretically significant features were noted. Thus, through a theory-led perspective on the initial data exploration, priority was given to theoretical significance and analytic parsimony. Accordingly, the features were *country* (*Kenya* dummy, *UK* dummy; the Thailand dummy was not needed in analytic model since Kenya = 0 and UK = 0 means Thailand = 1), *since_covid*, and *gender* (Male dummy; the Female dummy was not needed since Male = 0 means Female = 1). The outcome variable was *play_count* throughout model development.

**Fig. 2:**
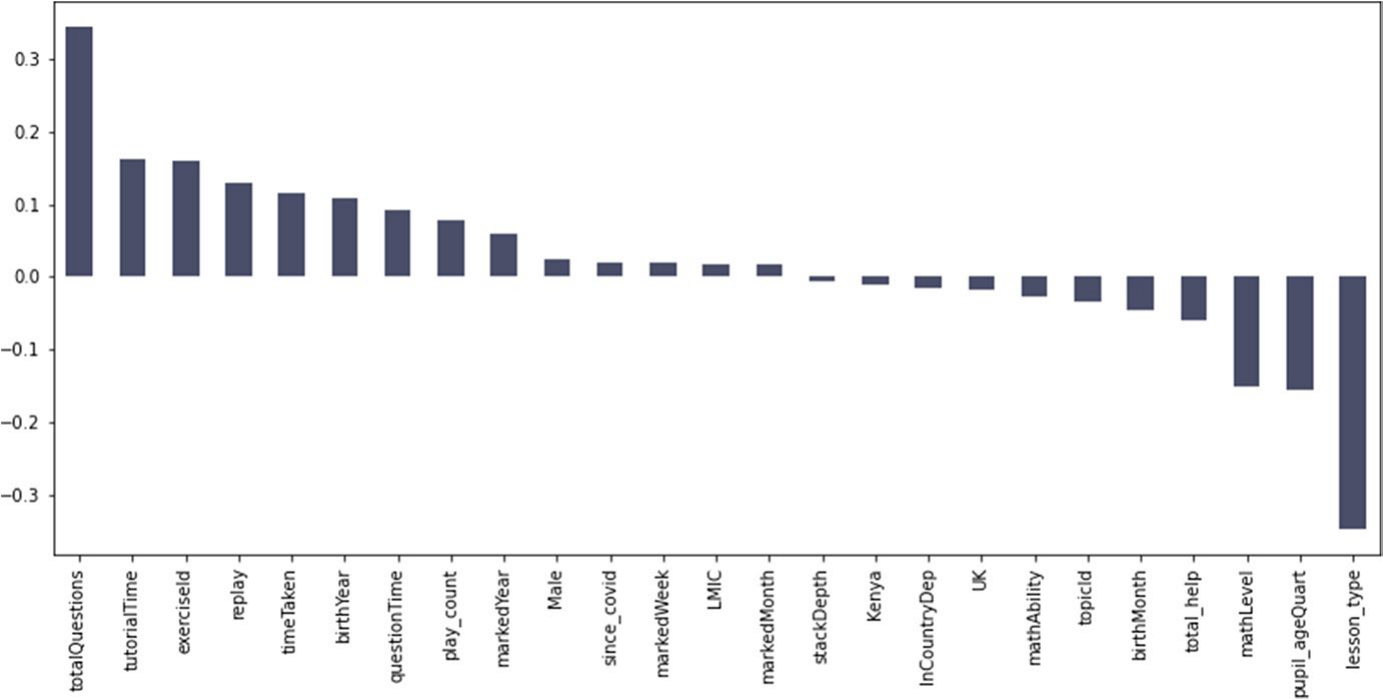
Correlations between potential features and learning outcome (*play_count*). Transformed data are represented here

Next, in Phase 2, the data-led approach to model development began with data-led feature selection. To begin with the variables available for feature selection excluded variables that represented the same construct as the target variable, *play_count*. Therefore, *indiv_pupil_t* was excluded. Also excluded were string variables such as marked and date of birth, which served as the bases of engineered features such as *marked_year* and *pupil_ageQuart*. The variables, Country and gender, were made redundant by use of the dummy variables relating to each: namely, *Kenya* and *UK* replaced country (again, Thailand was accounted for when Kenya = 0 and UK = 0) and *Male* replaced gender in the analytic model. Thus, 25 variables were available for feature selection during Phase 2, the data-led model development. These were: *topicId, mathLevel, exerciseId, stackDepth, timeTaken, questionTime, tutorialTime, totalQuestions, lesson_type, total_help, markedYear, markedMonth, markedWeek, Male, mathAbility, pupil_ageQuart, birthYear, birthMonth, replay, Kenya, UK, LMIC, since_covid, InCountryDep,* and *lesson_mark*. Again, the outcome variable was *play_count*. Correlations between learning outcomes (*play_count*) and the 25 potential features are shown in Fig. 2.

#### Outcomes

To report analytic insights from the final model, Shapley values were computed for interpretable feature importance, followed by Shapley interaction values in order to understand between-feature relationships (Aas et al., 2021; Rodríguez-Pérez & Bajorath, 2020). In doing so, analytic outcomes arising from the ‘black box’ of extreme gradient boosting can be mapped onto substantive concepts and enable theoretical contribution from the present research.

When appropriate, feature clusters are reported alongside individual feature importance analysis and feature interaction analyses. For this, hierarchical clustering is used, whereby features with distance = 0 are redundant (i.e., can replace the other[s] and the model still attains comparable performance [i.e., accuracy]) and those with distance = 1 are independent of each other (Lundberg & Lee, 2017).

### Analysis

The outcome variable was *play_count* throughout model development. Out of the full sample of n = 54,842,787, a random sample of n = 5,000 (random seed = 1) was used in analyses. Prior to model development, a baseline model was set up to predict *play_count*, after which model development commenced. In Phase 1, for the theory-led model development, the data frame containing only the theory-led features and the outcome variable was scaled, normalised, and missing data was imputed. In Phase 1, the theory-led model was resistant to improvement, with a persistent negative performance (i.e., training Adj R2) suggesting that a theory-only was insufficient: training RMSE = 654.29 and training Adj R^2^ = -13.45; the test RMSE = 601.58 and the test Adj R^2^ = -13.49 (for more details, see Appendix). A data-led approach to model development was necessary. In Phase 2, for the data-led model development, the data frame contained the data-led features and the outcome variable: these data were scaled, normalised, and imputed before data-led model development was conducted.

During both theory-led (Phase 1) and data-led (Phase 2) model development, simple linear regression models were run first. These were then regularised to adjust for non-linear features and distributions: grid search cross-validation (rather than randomised search cross-validation; Worcester, 2019) was used with elastic net penalty when elastic net cross validation revealed the Lasso and Ridge penalties on their own to be inappropriate, but that the combination of these (via the elastic net penalty) was required. Subsequently, extreme gradient boosting (a.k.a. XGBoost, Chen & Guestrin, 2016) was employed to maximise the computational resources available for peak speed and model performance (i.e., predictive performance). XGBoost models underwent automated hyperparameter tuning via grid search cross-validation (Worcester, 2019), followed by final manual hyperparameter tuning. The outcome of the model development, that is the final analytic model, is reported in the Appendix.

## Results

The analytic outcomes from the final model are now reported. Overall patterns of the final model are reported first, with plots presented in order of within-feature granularity. Analytic outcomes are then organised by features. The features that were identified for analysis through theory are reported first (i.e., features from the theory-led model; Hypotheses 1 to 3. The features that emerged as most important from data-led development are then reported upon (Hypothesis 4). Finally, feature interactions according to SHAP interaction values are examined. At times, figures will show subsamples of individual feature importance: these are then further subsampled from in the narrative, with particular focus on conceptual contribution to the field from the final model in this analysis.

### Overview of analytic outcomes

**Fig. 3:**
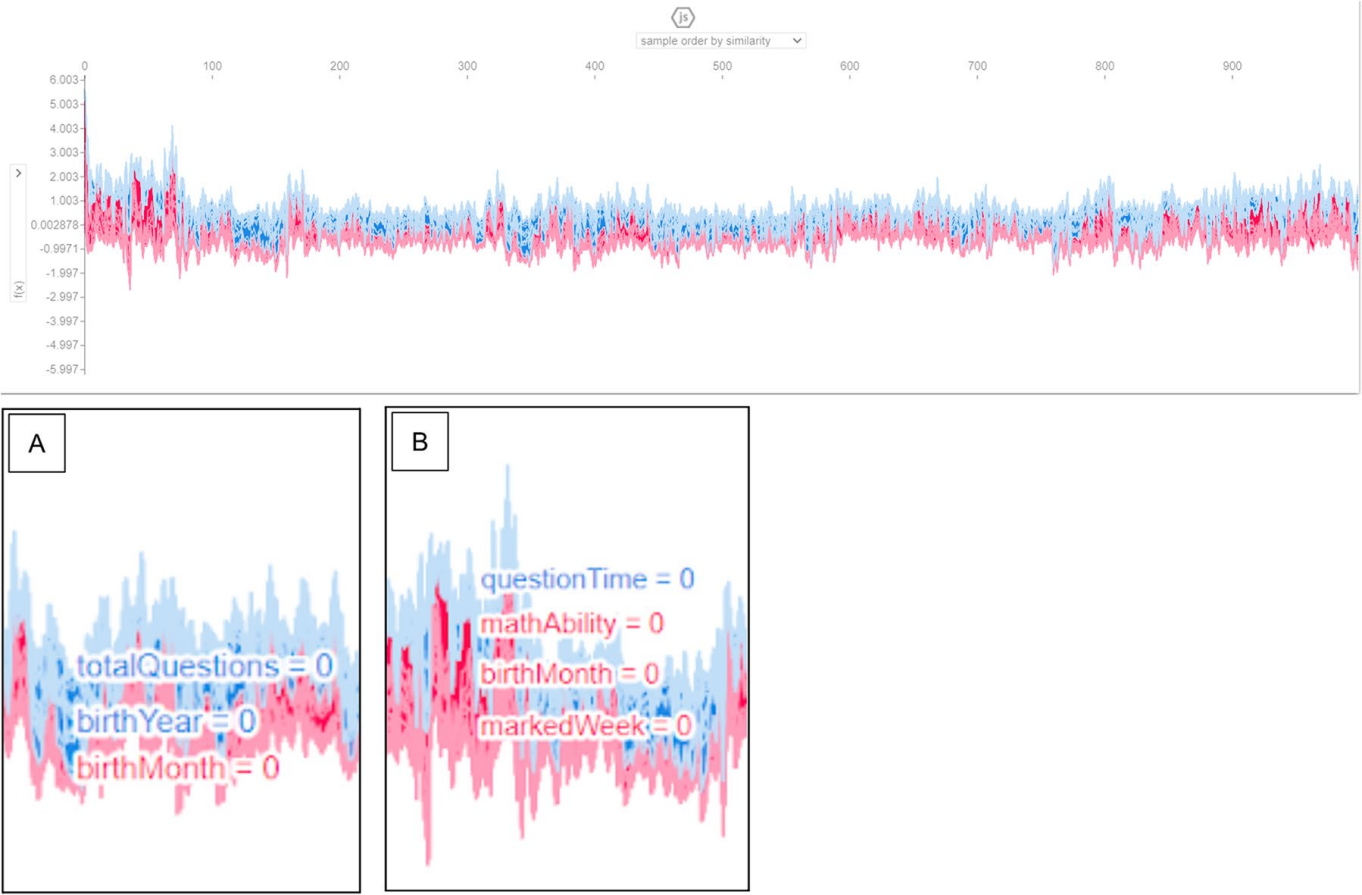
Collective force plot showing the overall effect of all features included in the final model, using absolute mean Shapley values. As the graph progresses to the right, effects of the most important features for each individual learner are shown. Features that push the prediction higher (to the right) are shown in red, and those pushing the prediction lower are in blue. The x-axis shows participant number, ordered by similarity for this plot. Panel **A** gives a snapshot of the features that generally reduce *play_count*; Panel **B** shows a snapshot of features that increase *play_count.* Transformed data are represented here

The collective force plot for the model (Fig. 3) shows that, among most learners, features in the final analytic model contribute to the decrease and decline of access to online learning (*play_count*), although some increase learning outcomes. Panel A suggests that the features potentially contributing to the decrease of access (*play_count*) include *totalQuestions* and *birthYear*. Meanwhile, Panel B shows *mathAbility, birthMonth*, and *markedWeek* to contribute to the increase of access. The subsequent analysis will shed more light on individual analyses.

**Fig. 4:**
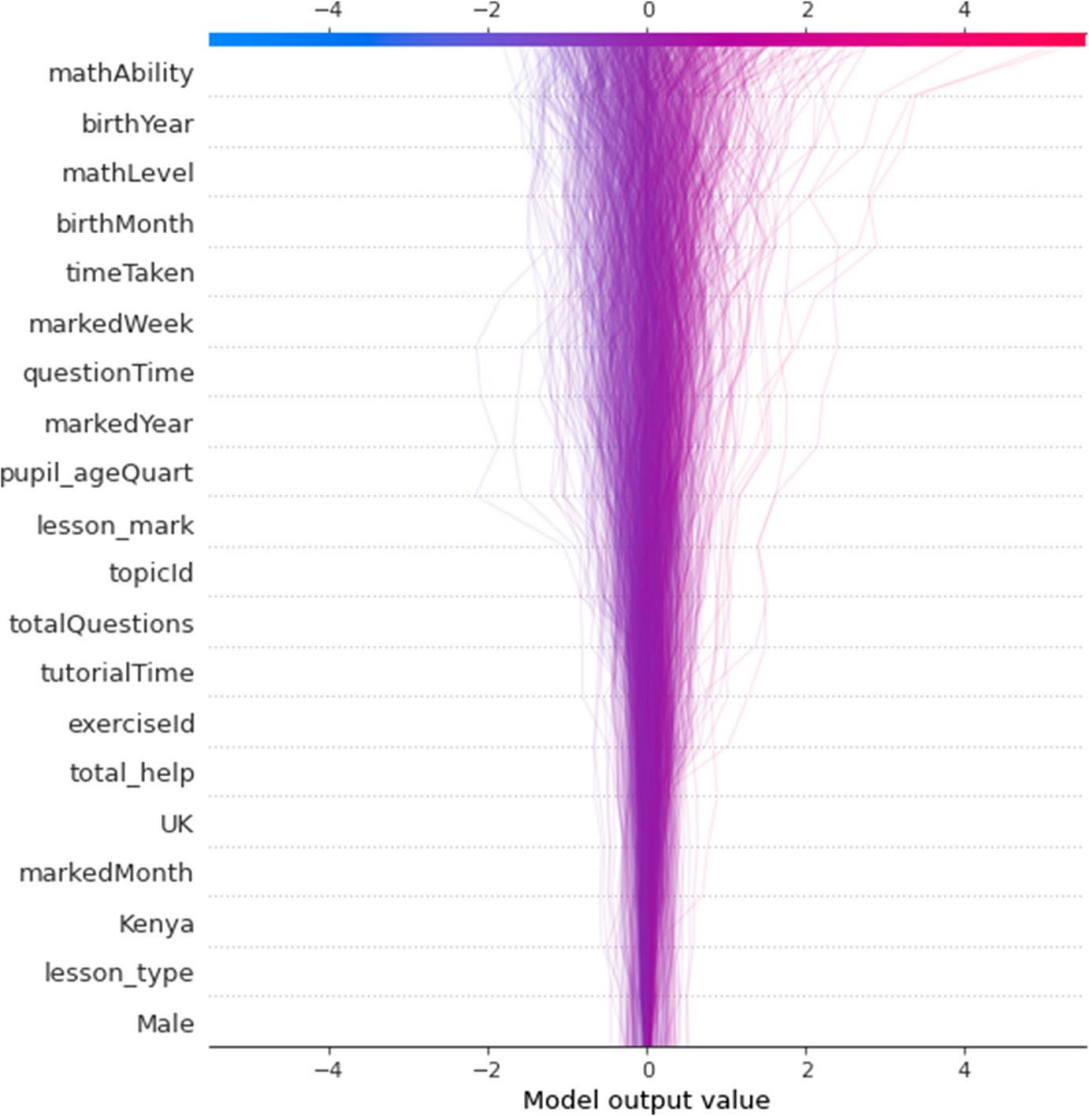
Decision plot of feature importance for global interpretation, using mean absolute Shapley values. The model output value is the learning outcome (*play_count*). Features that push the prediction higher (to the right) are shown in red, and those pushing the prediction lower are in blue. The fainter a line, the fewer learners it represents. Transformed data are represented here

The decision plot (Fig. 4) provides an overview of feature importance in the final model. It shows the distribution of feature importance and allows some between-feature comparison in terms of importance level and within-feature importance variability. There was reasonable homogeneity across included features in terms of within-feature importance variability, although heterogeneity increased somewhat with feature importance.

**Fig. 5:**
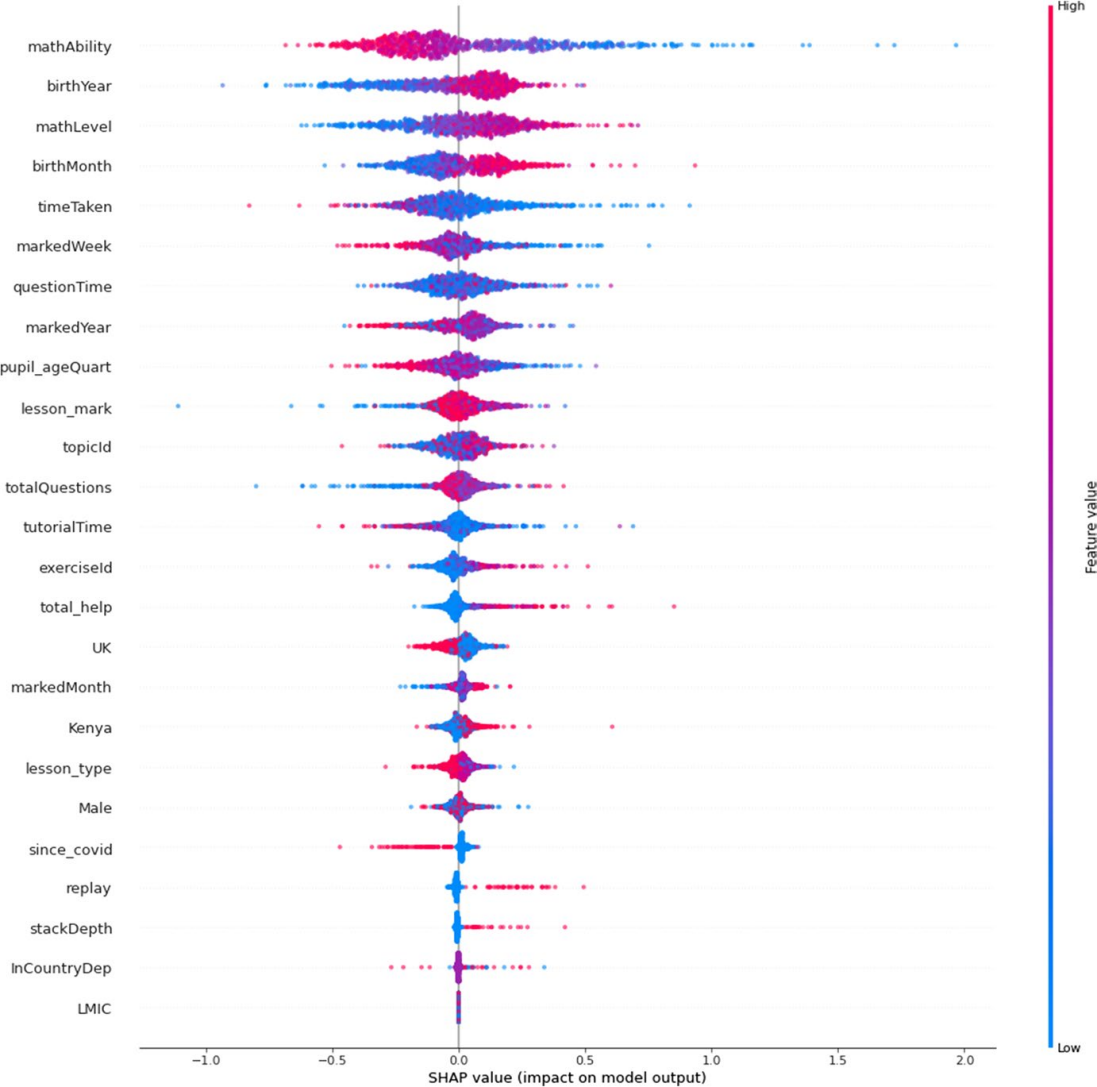
Summary plot of feature importance in final model, using mean absolute Shapley values. Features that push the prediction higher (to the right) are shown in red, and those pushing the prediction lower are in blue. Transformed data are represented here

Similarly, the summary plot (Fig. 5) shows the features in order of importance, but it provides greater granularity regarding the within-feature distribution of feature importance. Indeed, some of the most important feature, *mathAbility*, showed the greatest dispersion. Higher heterogeneity was also observed sporadically regardless of feature importance: this was shown by the features, *birthYear, birthMonth, timeTaken*, and *totalQuestions*.

### Individual feature analysis

Features emerging from the theoretical framework were those included in the theory-led model and will be examined first, followed by the most important features to emerge from the final model which was data-led. The means (M) and standard deviations (s.d.) reported are for the absolute Shapley values to emerge from the final, data-led XGBoost model.

#### Theory-led features

**Fig. 6:**
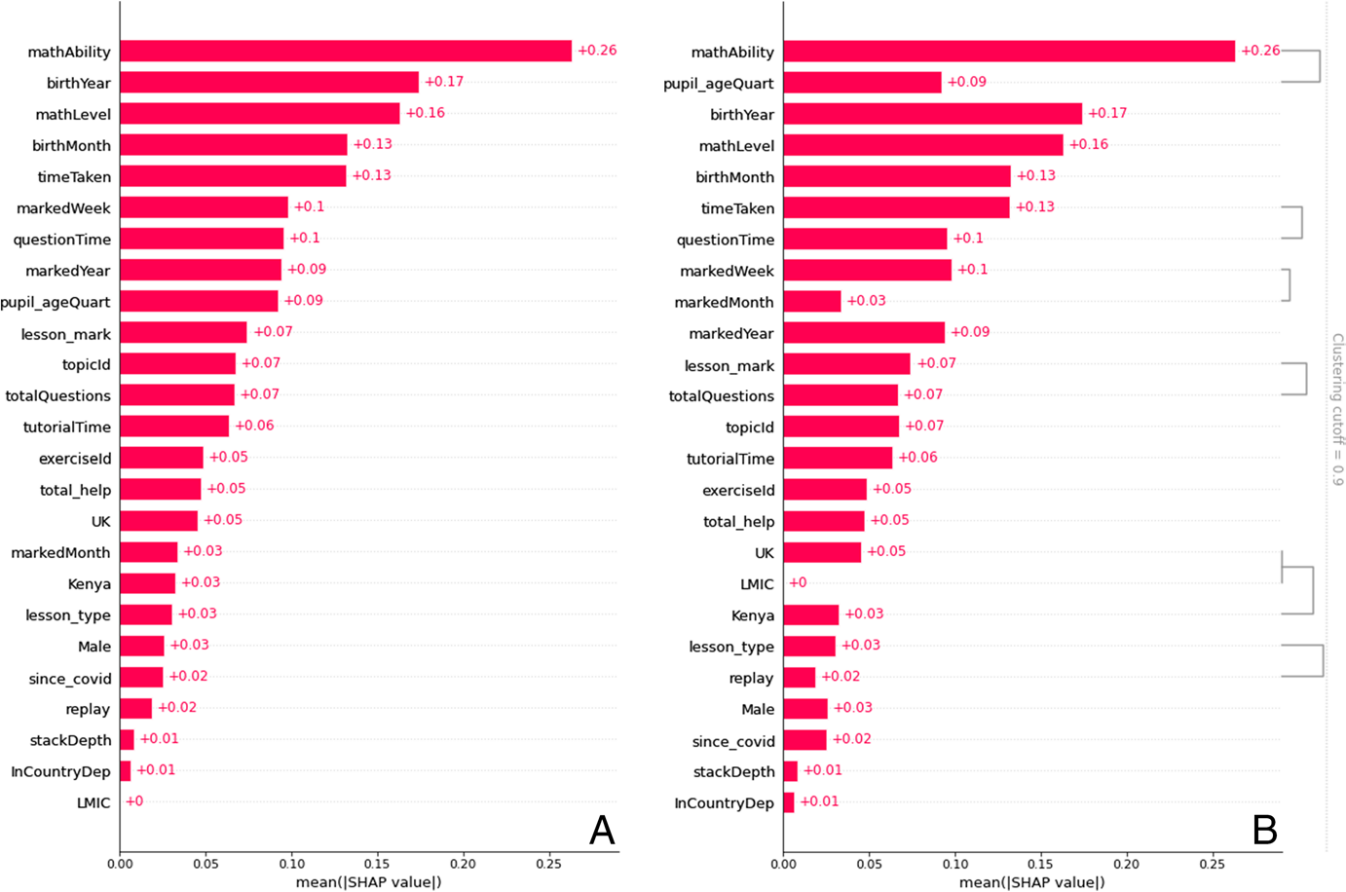
Bar plot of feature importance of features in the final model, using mean absolute Shapley values. Panel A shows the features ordered from the most important to the least, in the final model. Panel B shows the features are generally ordered in the same way, but with clustering where features are related to each other. Transformed data are represented here

**Fig. 7:**
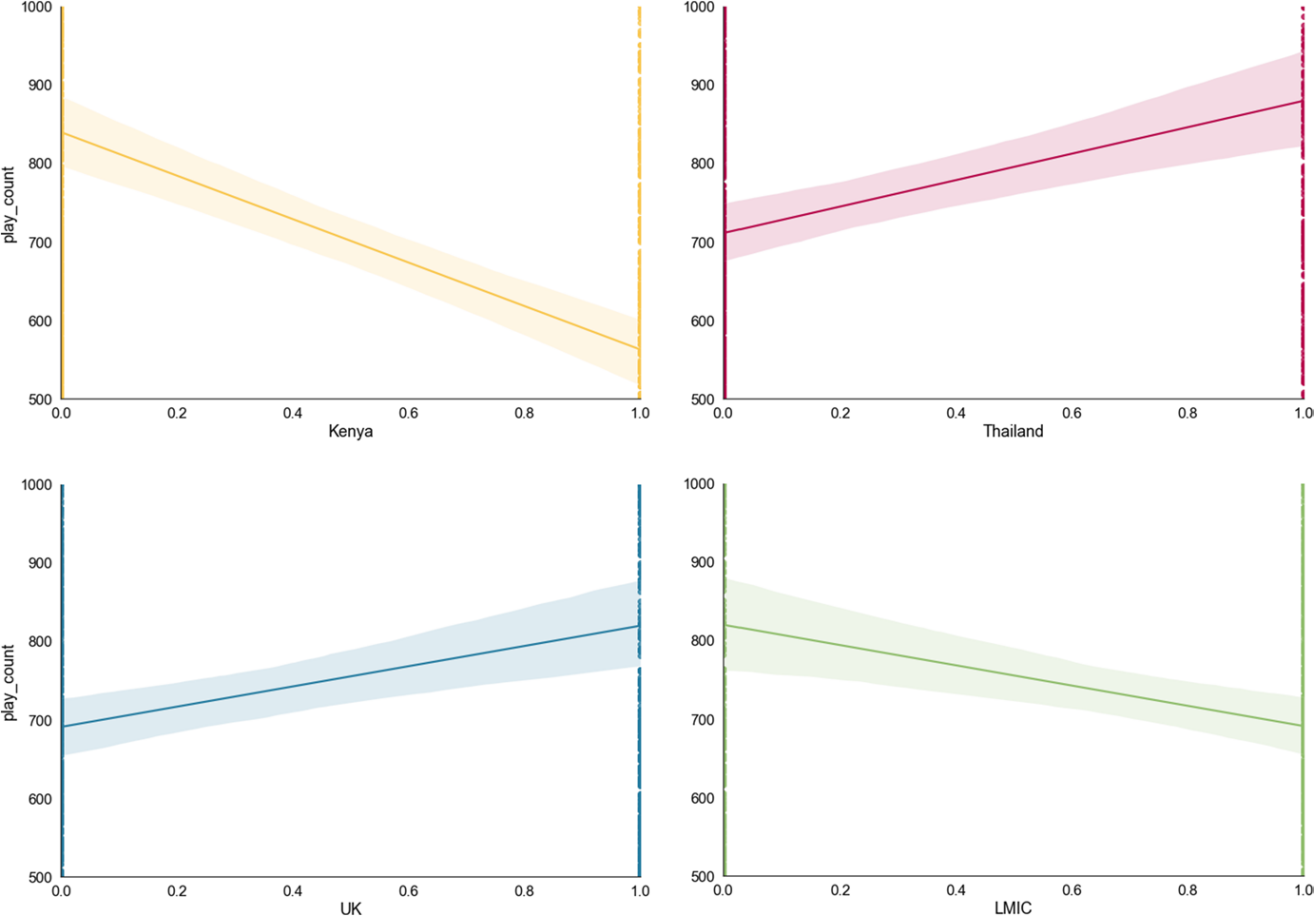
Line graphs showing how ‘country’ (Kenya, Thailand, and the UK) as well as LMIC status related to ‘access to online learning’ (*play_count*). Transformed data are represented here

From among the theory-led features, *country* was included in the final model (see Table 2, Features 6, 22, and 23) in accordance with the cross-validation with Elastic Net penalty. The *UK* was found to have the feature importance of absolute Shap M = 0.05 (s.d. = 0.03, Table 3); *Kenya* was also found to have the feature importance of absolute Shap M = 0.03 (s.d. = 0.04). Moreover, when each country is visually examined for their linearised relationship with *play_count*, Kenyan learners are found to be the most disadvantaged in terms of access to online learning (Fig. 7): whereas learners were less likely to access online learning if they were Kenyan, learners were more likely to have access if they were in Thailand or the UK. Furthermore, although *LMIC* (i.e., Kenya and Thailand) was found to have no feature importance on its own (absolute Shap M = 0.00, s.d. = 0.00), hierarchical clustering revealed *LMIC* to cluster with the country features, *Kenya* and *UK.* Thus, the hypothesised importance of country setting in access to online learning found some support in the final model, with some indication that countries’ LMIC status plays some role in predicting access to online learning (Fig. 6), as illustrated by the LMIC line graph (Fig. 7).

**Table 2:** Data-led model development. Coefficients (i.e., weights) that emerged from the regularised regression with the Elastic Net penalty when predicting access to online learning (*play_count*), in descending order of coefficient size

	Feature	Coefficient
1	mathAbility	-0.28722
2	InCountryDep	-0.14912
3	birthMonth	0.127049
4	birthYear	0.117863
5	mathLevel	0.110936
6	Kenya	-0.08101
7	exerciseId	0.066699
8	tutorialTime	-0.06467
9	total_help	0.056369
10	totalQuestions	0.054672
11	timeTaken	-0.05035
12	since_covid	0.049945
13	markedYear	0.045554
14	pupil_ageQuart	-0.04543
15	stackDepth	0.03957
16	markedWeek	-0.03924
17	topicId	0.032182
18	Male	0.02713
19	lesson_mark	0.019211
20	replay	0.018695
21	questionTime	-0.01032
22	UK	-0.00947
23	LMIC	0.008953
24	lesson_type	0.001756
25	markedMonth	0

**Table 3:** Shapley values for all the features in the final model for predicting *play_count*

	M	SD	min	max
mathAbility	0.263367	0.219041	0.001813	1.967418
birthYear	0.173876	0.136386	0.000677	0.933374
mathLevel	0.163275	0.128059	2.05E-05	0.709157
birthMonth	0.132435	0.098237	0.00017	0.93519
timeTaken	0.132227	0.126895	0.000171	0.913804
markedWeek	0.097876	0.102419	0.000113	0.752981
questionTime	0.095164	0.081499	3.77E-06	0.602196
markedYear	0.094058	0.076906	3.66E-05	0.452359
pupil_ageQuart	0.092162	0.085771	0.000123	0.543479
lesson_mark	0.074343	0.082897	8.61E-05	1.109786
topicId	0.067408	0.056775	4.13E-05	0.461668
totalQuestions	0.06661	0.088144	0.000104	0.800889
tutorialTime	0.063569	0.077716	8.35E-05	0.689795
exerciseId	0.048519	0.051968	1.13E-05	0.511054
total_help	0.047218	0.075228	2.12E-05	0.85206
UK	0.045384	0.034569	3.30E-06	0.198564
markedMonth	0.033839	0.032279	6.75E-05	0.23072
Kenya	0.032443	0.03541	0.000127	0.607252
lesson_type	0.030549	0.028919	2.24E-05	0.28909
Male	0.02577	0.028436	2.99E-05	0.275197
since_covid	0.02499	0.043463	6.24E-05	0.469803
replay	0.018683	0.046888	6.79E-05	0.4945
stackDepth	0.008345	0.02338	0.000152	0.420604
InCountryDep	0.006133	0.024946	6.03E-07	0.338753
LMIC	0	0	0	0

**Fig. 8:**
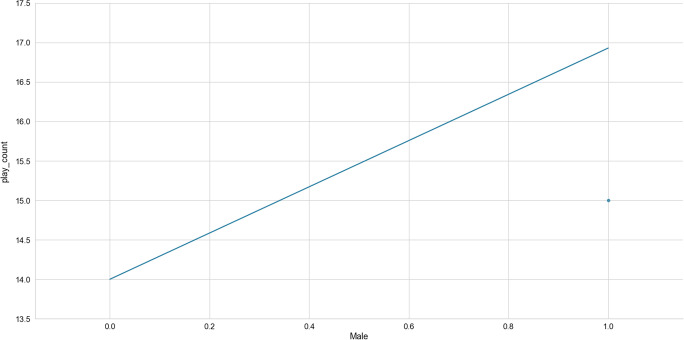
The role of gender (*Male*, dummy variable) in predicting access to online learning (*play_count*)

The expected gender effect found support as the feature, *Male,* was included in the final model (Table 2, feature 18). The feature, *Male,* was found to have some importance, according to the absolute Shap M = 0.03, s.d. = 0.03. As can be seen in Fig. 8, boys are more likely to access online learning than girls.

**Fig. 9:**
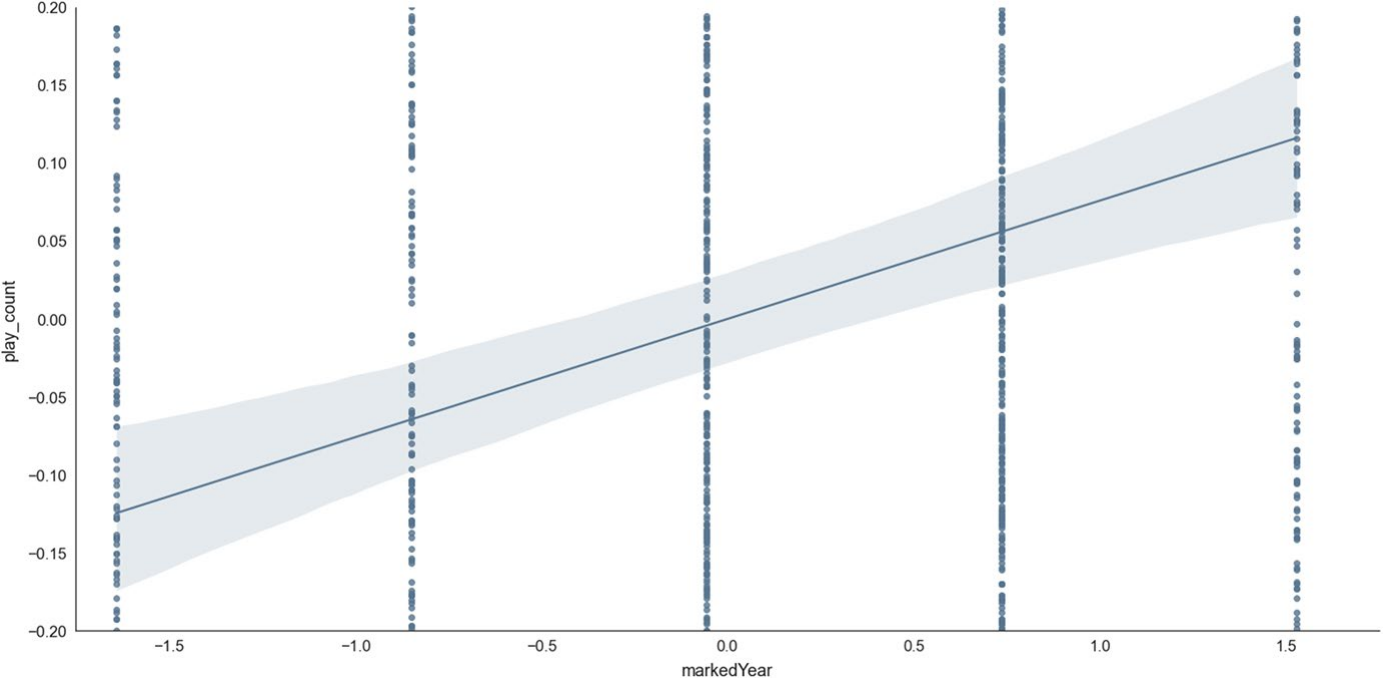
Access to online learning (*play_count*) as the years (*markedYear*) progress, with the final time point representing the year 2020 (i.e., from the onset of Covid). Transformed data are represented here

The anticipated Covid effect was seen in the final model via the primary feature, *since_covid*, qualifying to be selected as a feature in the final model (Table 2, Feature 12). This emerged to have the importance of absolute Shap M = 0.02, s.d. = 0.04. Additionally, *markedYear* was a related feature for the same concept which also qualified in feature selection (Table 2, Feature 13), emerging with some importance (absolute Shap M = 0.09, s.d. = 0.08). Together, the two features offered support to the hypothesised importance of COVID-19 in predicting online learning outcomes (Fig. 9).

#### Data-led features

**Fig. 10:**
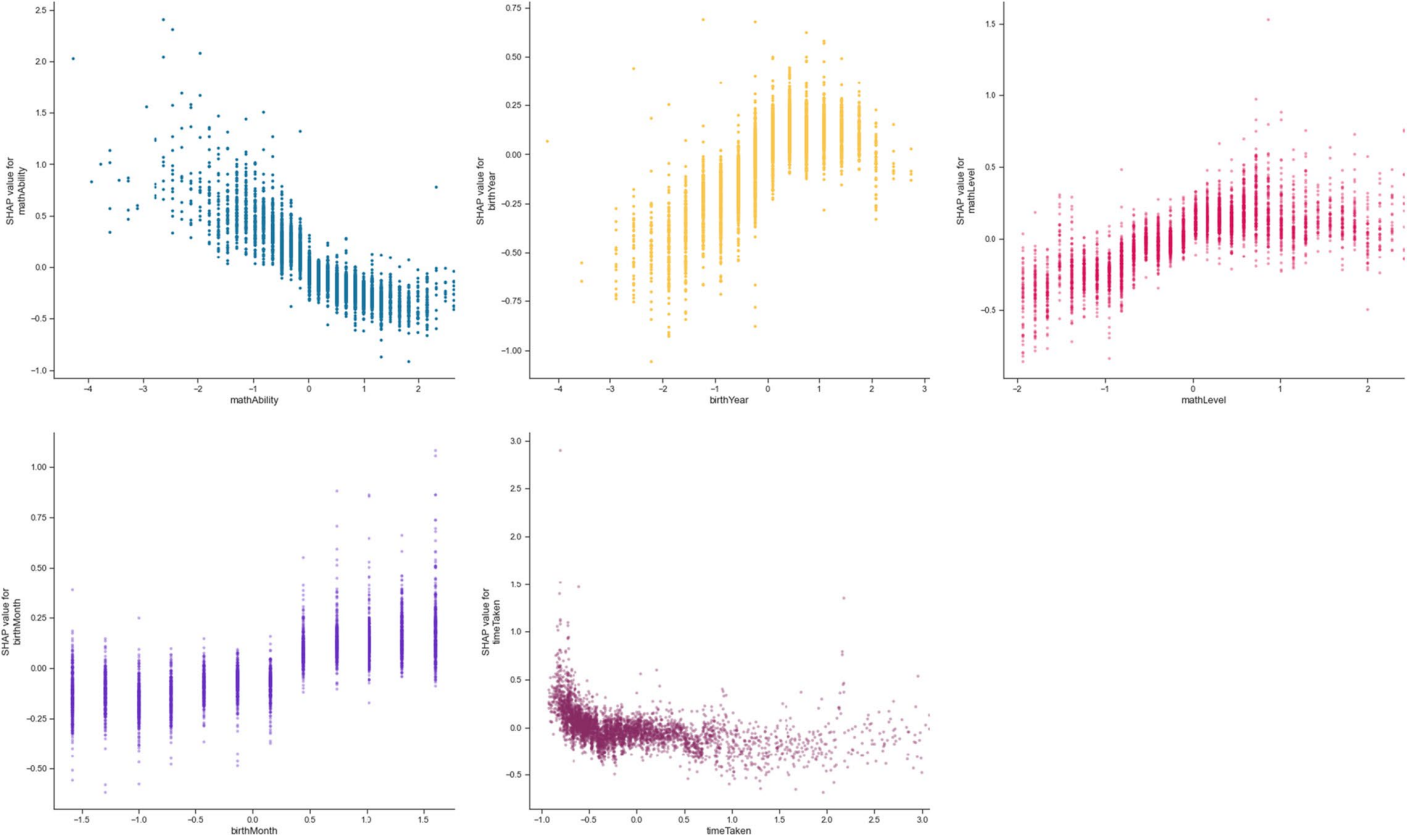
Top five most important features in predicting online learning access (*play_count*). Transformed data are represented here

**Fig. 11:**
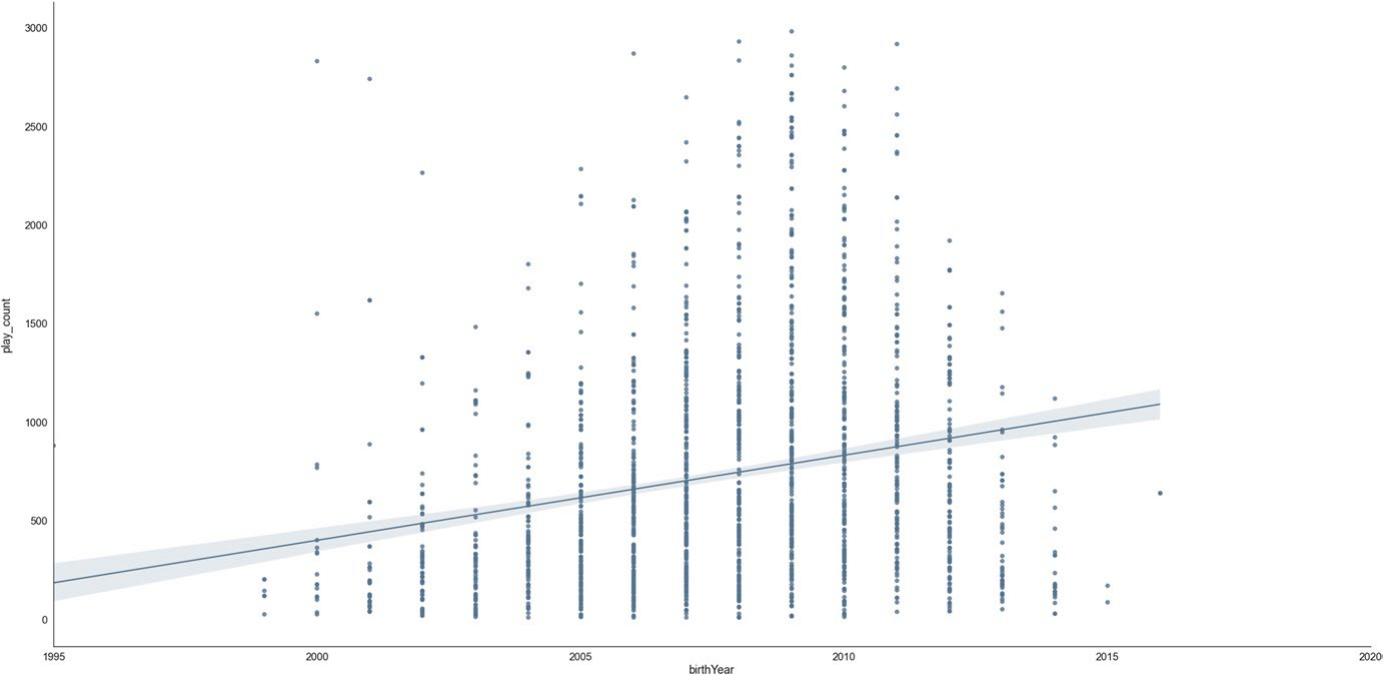
Scatter plot showing how *birthYear* was related to online learning access (*play_count*). Untransformed data are represented here

Based on the final, data-led model, the most important features include *mathAbility* (absolute Shap M = 0.36, s.d. = 0.22)*, birthYear* (absolute Shap M = 0.17, s.d. = 0.14)*, mathLevel* (absolute Shap M = 0.16, s.d. = 0.13)*, birthMonth* (absolute Shap M = 0.13, s.d. = 0.10)*,* and *timeTaken* (absolute Shap M = 0.13, s.d. = 0.13)*.* Figure 10 shows these five most important features in predicting access to online learning. It shows the relationship between access to online learning *(play_count*) and *mathAbility*: there appears to be an optimal maths ability level, after which maths ability declines with *play_count*. Additionally, the older the learner (*birthYear*), the higher their *play_count* ー although those born from and after 2010 (i.e., aged 10 and younger) were decreasingly likely to return to this online learning platform (Figs. 10 and 11). Access was found to increase with *mathLevel* (i.e. difficulty) of the lesson (Fig. 10). Learners born between August and December (inclusive) were more likely to access online learning, whereas learners born in other months were less likely to access the online platform (Fig. 10). Finally, the less time the learner took to complete each lesson, the less they accessed the online platform (Fig. 10).

### Feature interactions

**Fig. 12:**
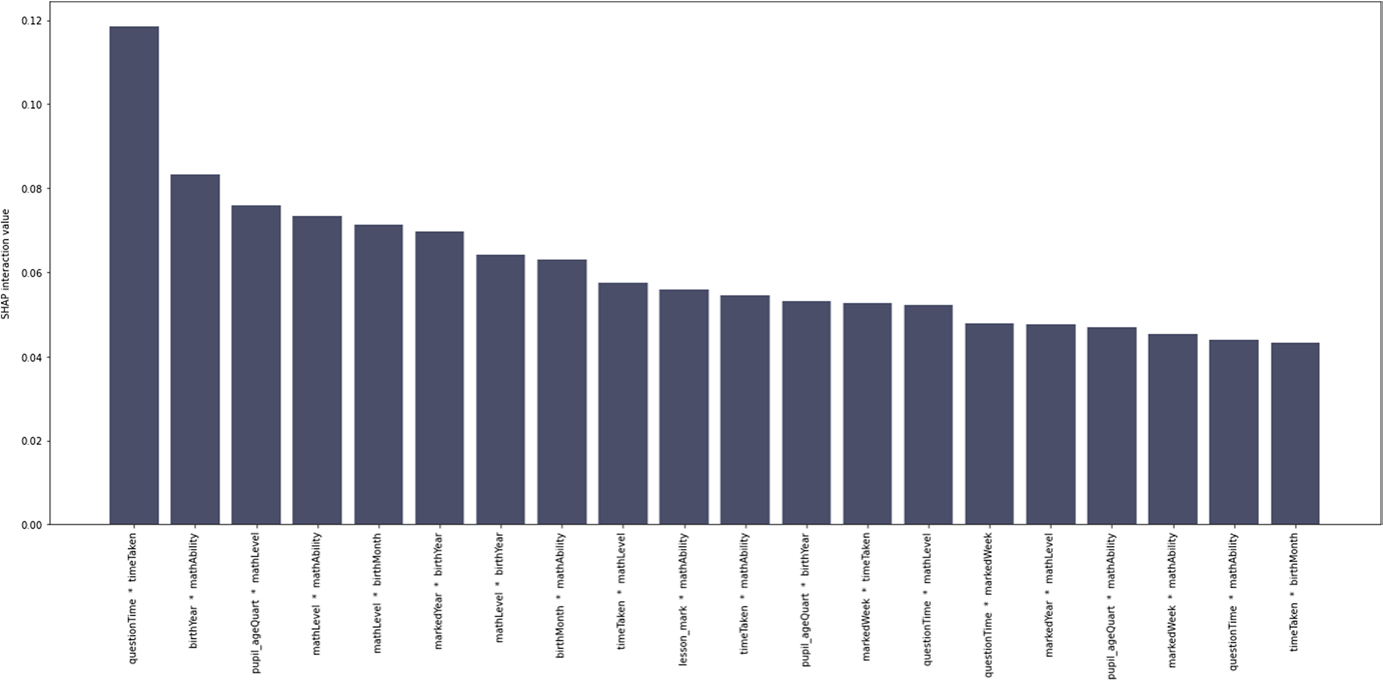
SHAP interaction values for predicting access to online learning (*play_count*), from the strongest interaction to the weakest. Only the top 20 interactions are shown here. Transformed data are represented here

Table 4 shows the top 70 feature interaction pairs. Figure 12 shows the top 20 interactions. However, the remainder of the Results section will discuss the features that interacted with the theory-led features in the final model, followed by the top six interacting feature pairs which are also those with Shap interaction values (ϕ) of 0.07 and above (Table 4).

**Table 4:** The top 70 SHAP interaction values

	Feature	Shap interaction value	cum_diff
1	questionTime * timeTaken	0.12	NA
2	birthYear * mathAbility	0.08	-0.04
3	pupil_ageQuart * mathLevel	0.08	-0.01
4	mathLevel * mathAbility	0.07	0
5	mathLevel * birthMonth	0.07	0
6	markedYear * birthYear	0.07	0
7	mathLevel * birthYear	0.06	-0.01
8	birthMonth * mathAbility	0.06	0
9	timeTaken * mathLevel	0.06	-0.01
10	lesson_mark * mathAbility	0.06	0
11	timeTaken * mathAbility	0.05	0
12	pupil_ageQuart * birthYear	0.05	0
13	markedWeek * timeTaken	0.05	0
14	questionTime * mathLevel	0.05	0
15	questionTime * markedWeek	0.05	0
16	markedYear * mathLevel	0.05	0
17	pupil_ageQuart * mathAbility	0.05	0
18	markedWeek * mathAbility	0.05	0
19	questionTime * mathAbility	0.04	0
20	timeTaken * birthMonth	0.04	0
21	markedYear * mathAbility	0.04	0
22	timeTaken * tutorialTime	0.04	0
23	lesson_mark * totalQuestions	0.04	0
24	pupil_ageQuart * timeTaken	0.04	0
25	questionTime * pupil_ageQuart	0.04	0
26	lesson_mark * birthYear	0.04	0
27	questionTime * birthMonth	0.04	0
28	UK * birthYear	0.04	0
29	markedWeek * mathLevel	0.04	0
30	total_help * tutorialTime	0.04	0
31	topicId * mathAbility	0.04	0
32	timeTaken * totalQuestions	0.04	0
33	topicId * timeTaken	0.03	0
34	birthYear * birthMonth	0.03	0
35	topicId * birthYear	0.03	0
36	timeTaken * birthYear	0.03	0
37	markedWeek * birthMonth	0.03	0
38	tutorialTime * mathAbility	0.03	0
39	lesson_mark * mathLevel	0.03	0
40	questionTime * topicId	0.03	0
41	pupil_ageQuart * birthMonth	0.03	0
42	tutorialTime * birthMonth	0.03	0
43	lesson_mark * timeTaken	0.03	0
44	totalQuestions * tutorialTime	0.03	0
45	markedWeek * pupil_ageQuart	0.03	0
46	pupil_ageQuart * markedYear	0.03	0
47	markedWeek * birthYear	0.03	0
48	Kenya * mathLevel	0.03	0
49	totalQuestions * mathAbility	0.03	0
50	markedYear * birthMonth	0.03	0
51	tutorialTime * mathLevel	0.03	0
52	timeTaken * total_help	0.03	0
53	lesson_mark * birthMonth	0.03	0
54	topicId * mathLevel	0.03	0
55	questionTime * tutorialTime	0.03	0
56	Male * timeTaken	0.03	0
57	questionTime * totalQuestions	0.03	0
58	markedWeek * totalQuestions	0.03	0
59	questionTime * birthYear	0.03	0
60	markedWeek * tutorialTime	0.03	0
61	markedWeek * markedYear	0.03	0
62	UK * mathLevel	0.03	0
63	questionTime * lesson_mark	0.03	0
64	lesson_mark * topicId	0.03	0
65	timeTaken * exerciseId	0.03	0
66	topicId * markedWeek	0.02	0
67	lesson_mark * markedWeek	0.02	0
68	topicId * Kenya	0.02	0
69	topicId * pupil_ageQuart	0.02	0
70	markedWeek * exerciseId	0.02	0

#### Interactants with theory-led features

**Fig. 13:**
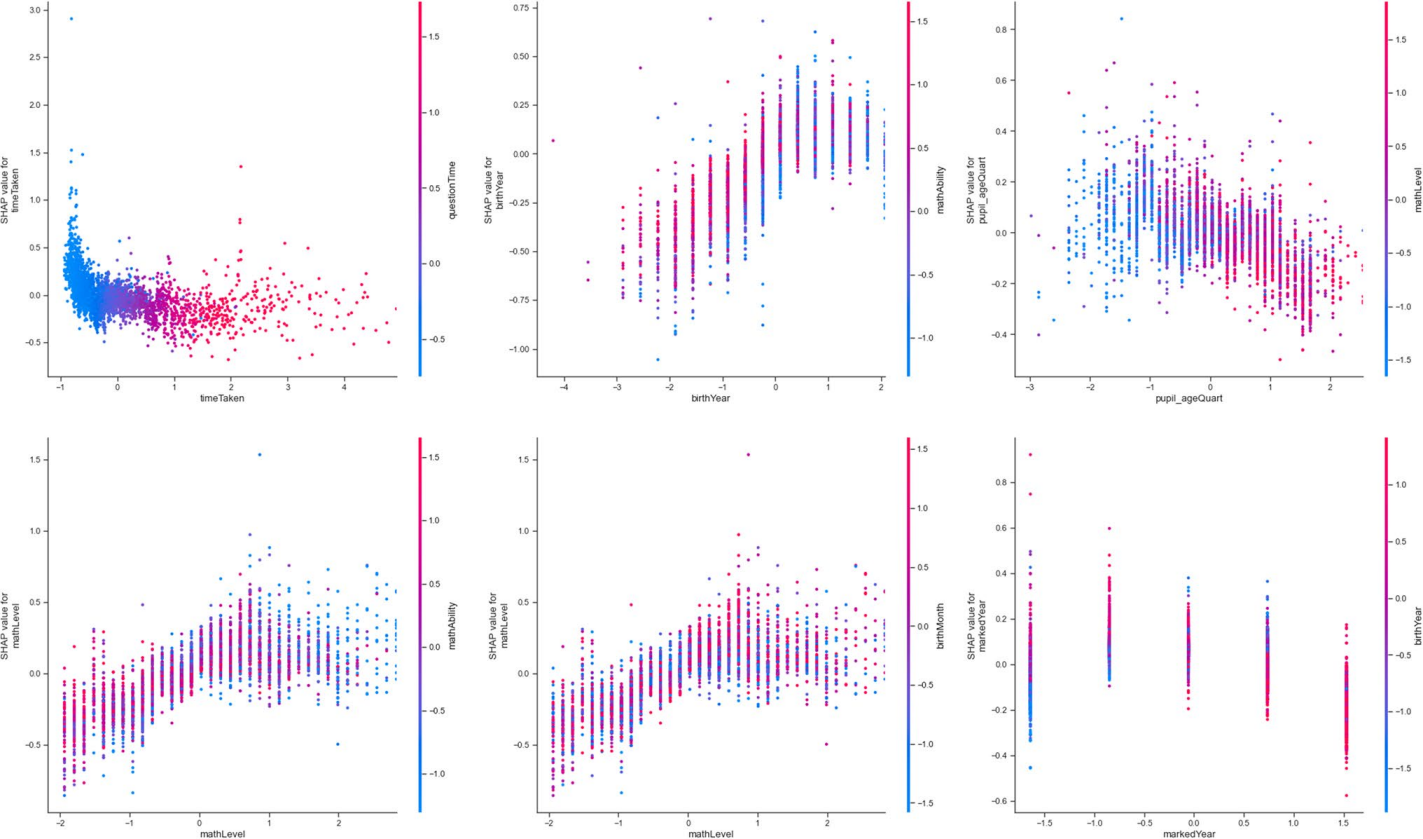
Dependence plots for the six strongest interactants to emerge from the final model

**Fig. 14:**
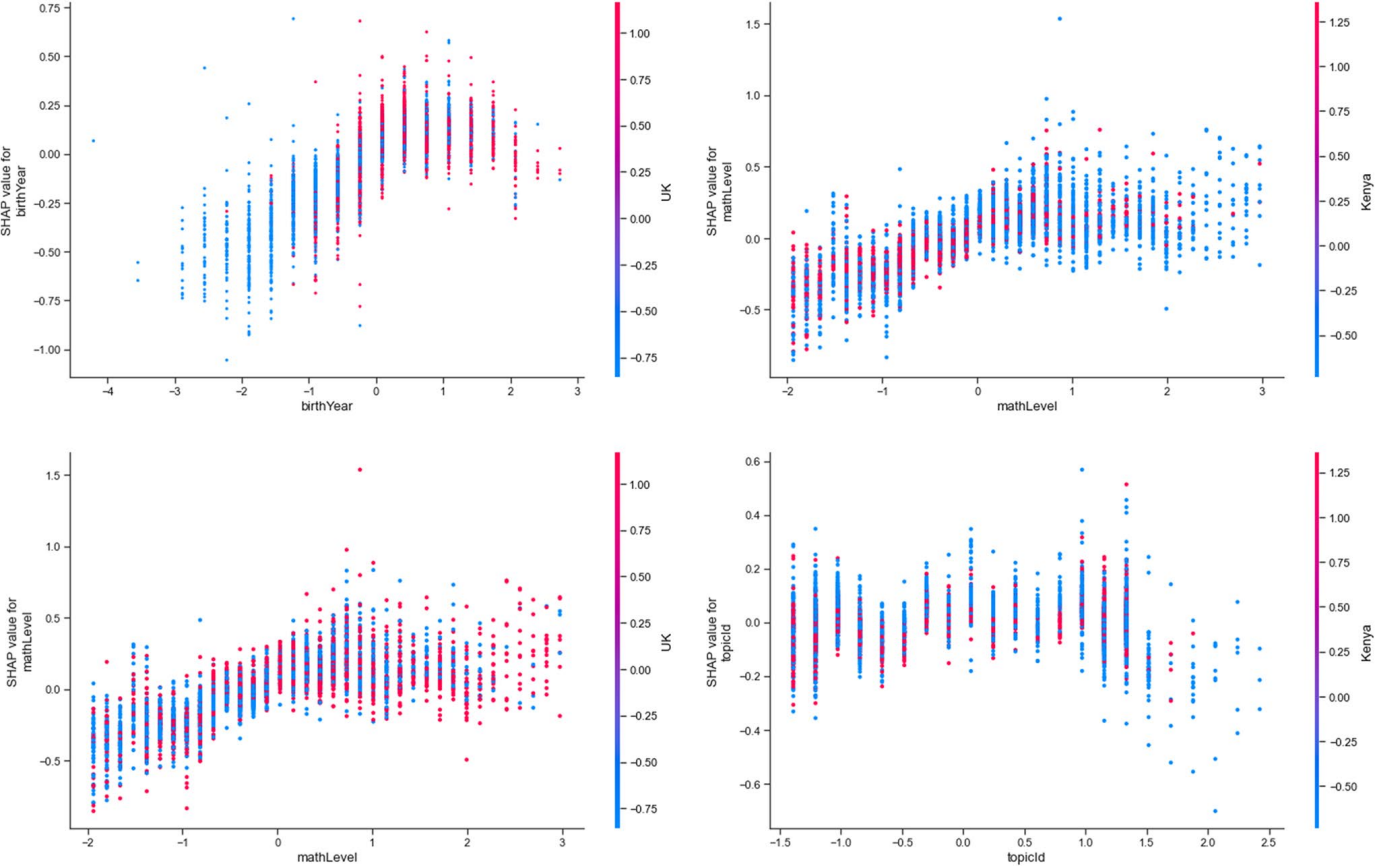
Dependence plots of the importance of the features that interact with Country (either UK or Kenya) in predicting learning outcomes (lesson mark), according to mean absolute Shapley values. Transformed data are represented here

Upon inspecting the top 70 interacting feature pairs (Table 4), country features could be found to form 6 interacting features pairs (ϕ ⋝ 0.02). These are shown in Fig. 13. Among these, UK particularly combines with *birthYear* to push learning outcomes lower (ϕ = 0.04): that is, the younger the learners, the lower the *play_count,* in the UK; however, the younger the learners, the higher the *play_count* outside of the UK (Fig. 14). Thus, there is a suggestion of decreasing access to online learning with learner age in LMIC settings (i.e., Kenya and Thailand). A complementary pattern was observed in country’s interaction with lesson difficulty (*mathLevel*): access to online learning increased with lesson difficulty, but only among Thai learners; access decreased with lesson difficulty among UK (ϕ = 0.03) and Kenyan (ϕ = 0.03) learners. This suggests a cultural effect on educational access, whereby Thai learners are given more access with lesson difficulty. Also, learners accessed lessons with differing *topicId* depending on their country settings, suggesting between-country differences in the relevance of each topic (ϕ = 0.02).

**Fig. 15:**
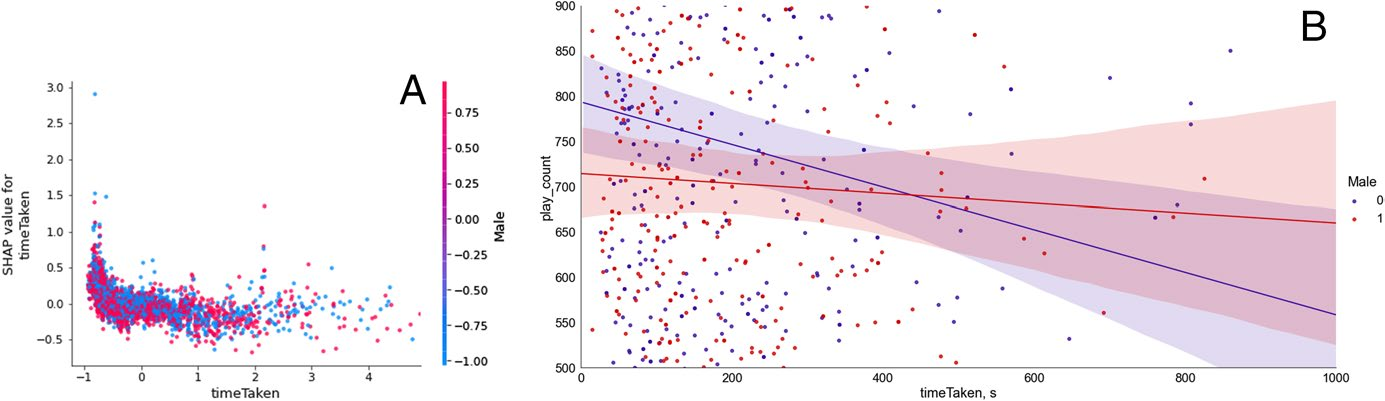
The interaction between gender (*Male*) and *timeTaken* to complete each lesson. Panel **A** represents transformed data and relates to feature importance via absolute Shapley values; Panel **B** represents untransformed data and reflects associations between the variables

Gender (*Male*) interacted with one other feature in predicting access to online learning (*play_count*): namely, the *timeTaken* to complete a lesson (Fig. 15). Whereas boys continued to access online learning regardless of the time they took to complete a lesson, girls’ access to online learning decreased as the *timeTaken* to complete a lesson increased.

**Fig. 16:**
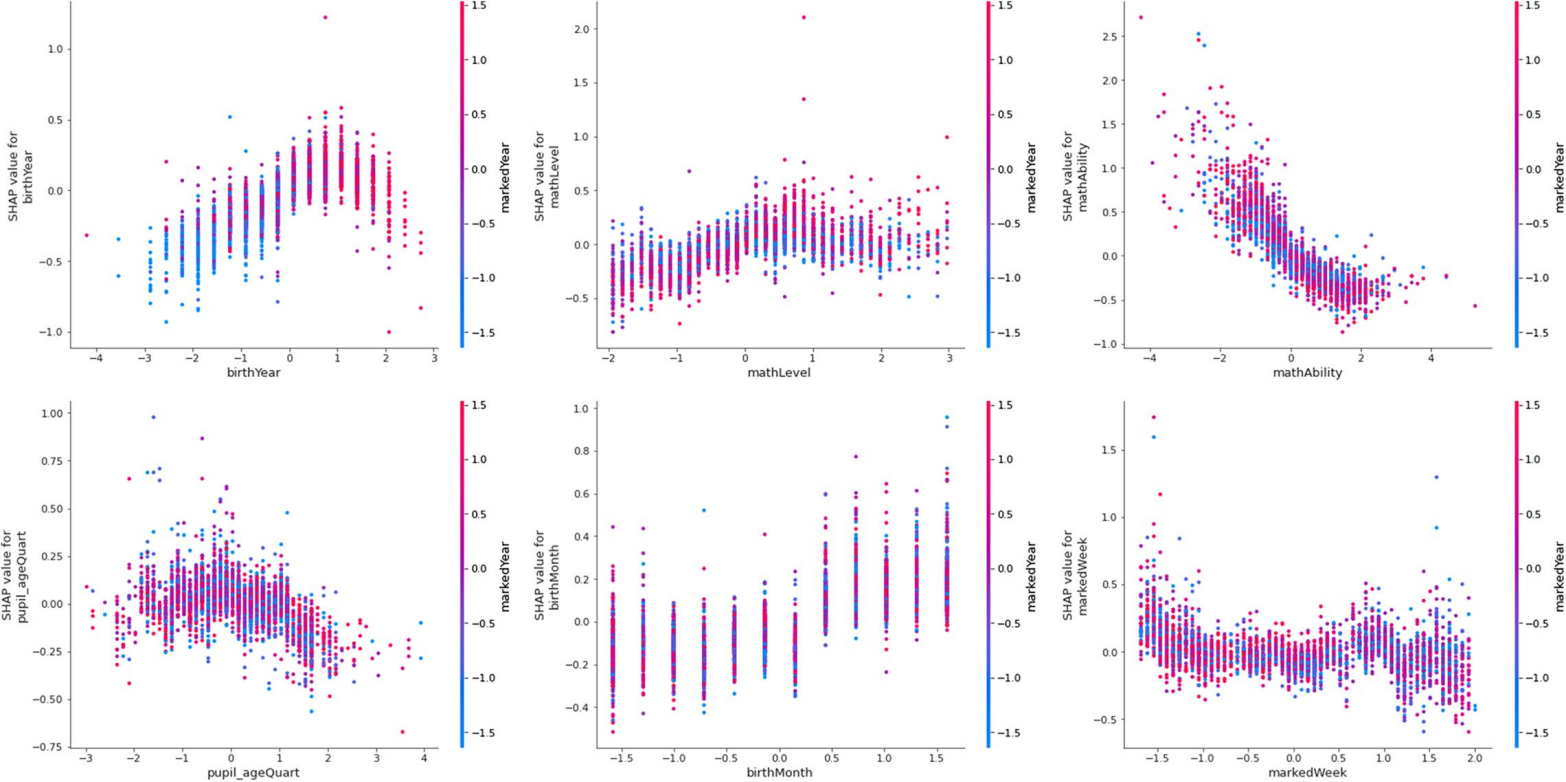
Dependence plots of the importance of the features that interact with Covid, as measured by *markedYear,* in predicting access to online learning (*play_count*), according to mean absolute Shapley values. Transformed data are represented here

**Fig. 17:**
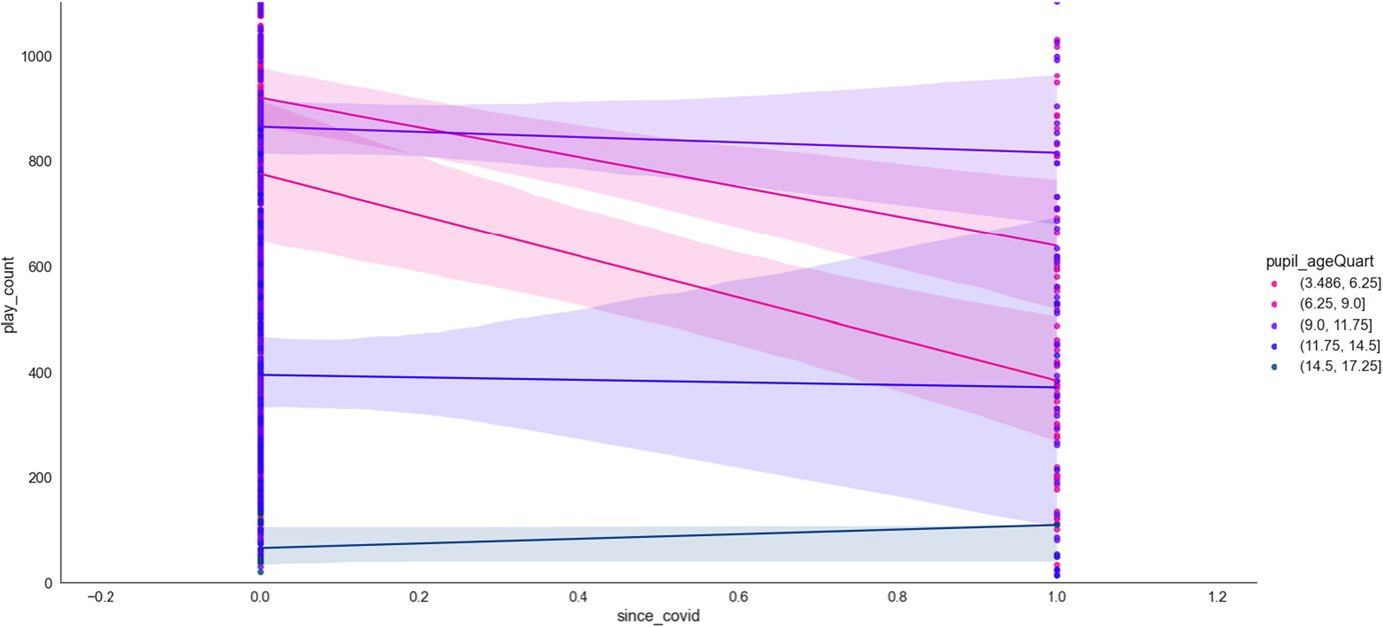
The interaction between learner age (*pupil_ageQuart*) and Covid (i.e., *markedYear*; pre-covid = 2015–2019, since covid = 2020 onwards) in predicting access to online learning. Untransformed data are represented here

COVID-19 (as measured by *markedYear*) could be found to form six interacting features pairs when predicting access to online learning (*play_count*, ϕ ⋝ 0.03). From Table 4, *markedYear* could be found to predict access with three potential interactants (see also Fig. 16). Access increased as age (*birthYear*) decreased pre-Covid, but access decreased with age post-Covid (ϕ = 0.07). Younger learners (*pupil_ageQuart*) access online learning less post-Covid, whereas older learners are unaffected (see also Fig. 17; ϕ = 0.03).

**Fig. 18:**
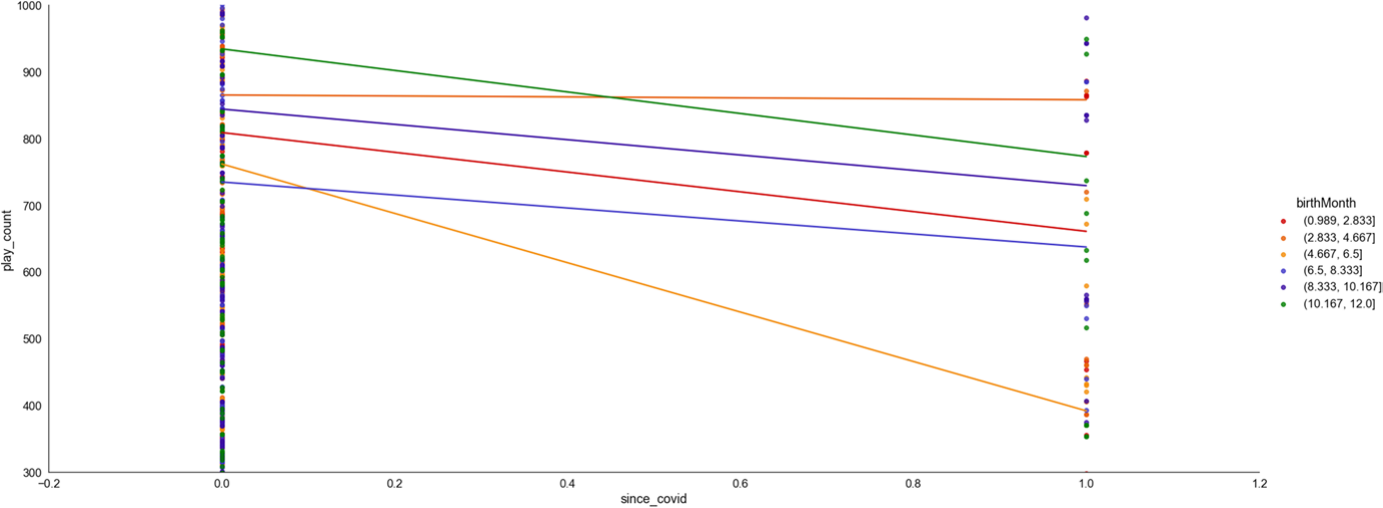
The interaction between *birthMonth* and Covid (i.e., *markedYear*; pre-covid = 2015–2019, since covid = 2020 onwards) in predicting access to online learning (*play_count*). Untransformed data are represented here

Access decreased with lesson difficulty (*mathLevel*) pre-Covid, but access increases with lesson difficulty post-Covid (ϕ = 0.05). Access increased with *mathAbility* pre-Covid, but access decreases as *mathAbility* increases post-Covid (ϕ = 0.04). Those born (*birthMonth*) in the first half of the year and in the final two months of the year suffered from decreased access to online learning post-Covid, whereas others’ access was unaffected by COVID-19 (see also Fig. 18; ϕ = 0.03).

**Fig. 19:**
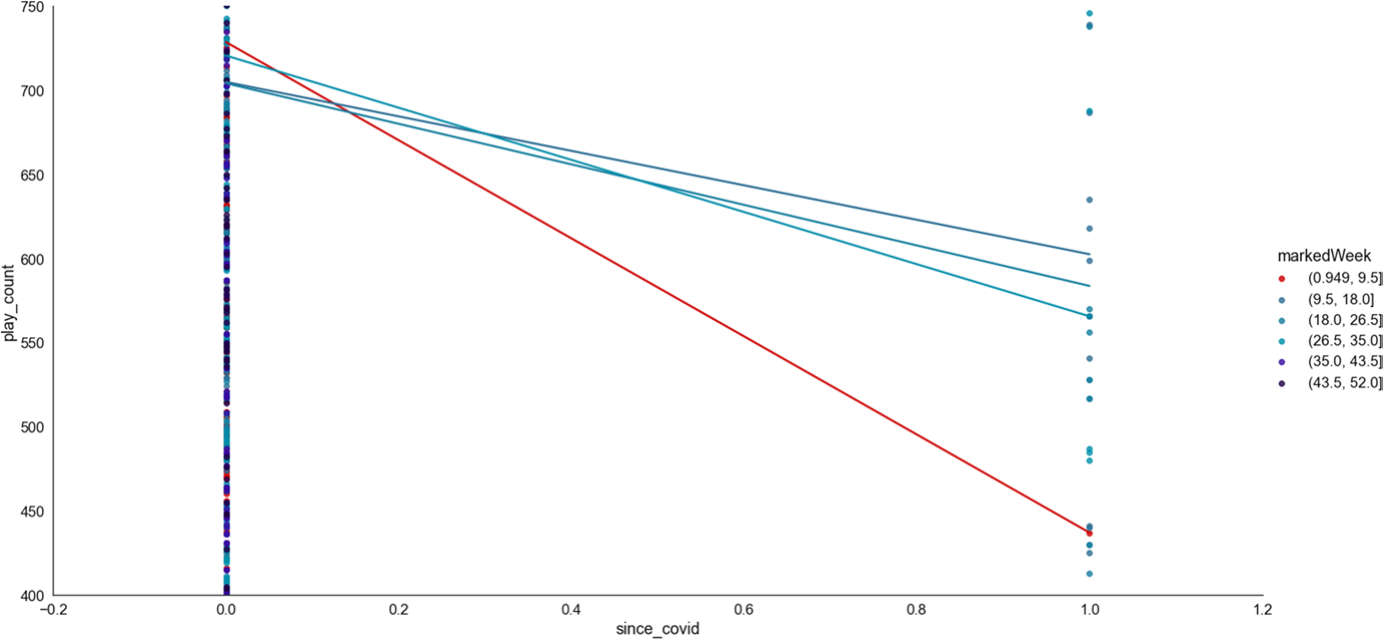
The interaction between markedWeek and Covid (i.e., *markedYear*; pre-covid = 2015–2019, since covid = 2020 onwards) in predicting access to online learning (*play_count*). Untransformed data are represented here

The first eight or so weeks (*markedWeek*) in the year saw a significant decline in online learning when COVID-19 began, but the remaining weeks in the year saw no change due to COVID-19 (see also Fig. 19, ϕ = 0.03). Thus, COVID-19 increased access to online learning when combined with lesson difficulty (*mathLevel*). However, access was reduced when COVID-19 combined with younger learner age (*pupil_ageQuart*), learners born at the start and end of the year (*birthMonth*), and when online learning occurred during the first eight weeks of the calendar year (*markedWeek*).

In all, support was added to the theory-led features for explaining access to online learning and interactants were uncovered to add insight into the way country, COVID-19, and gender explain access to online learning.

#### Data-led feature interactions

**Fig. 20:**
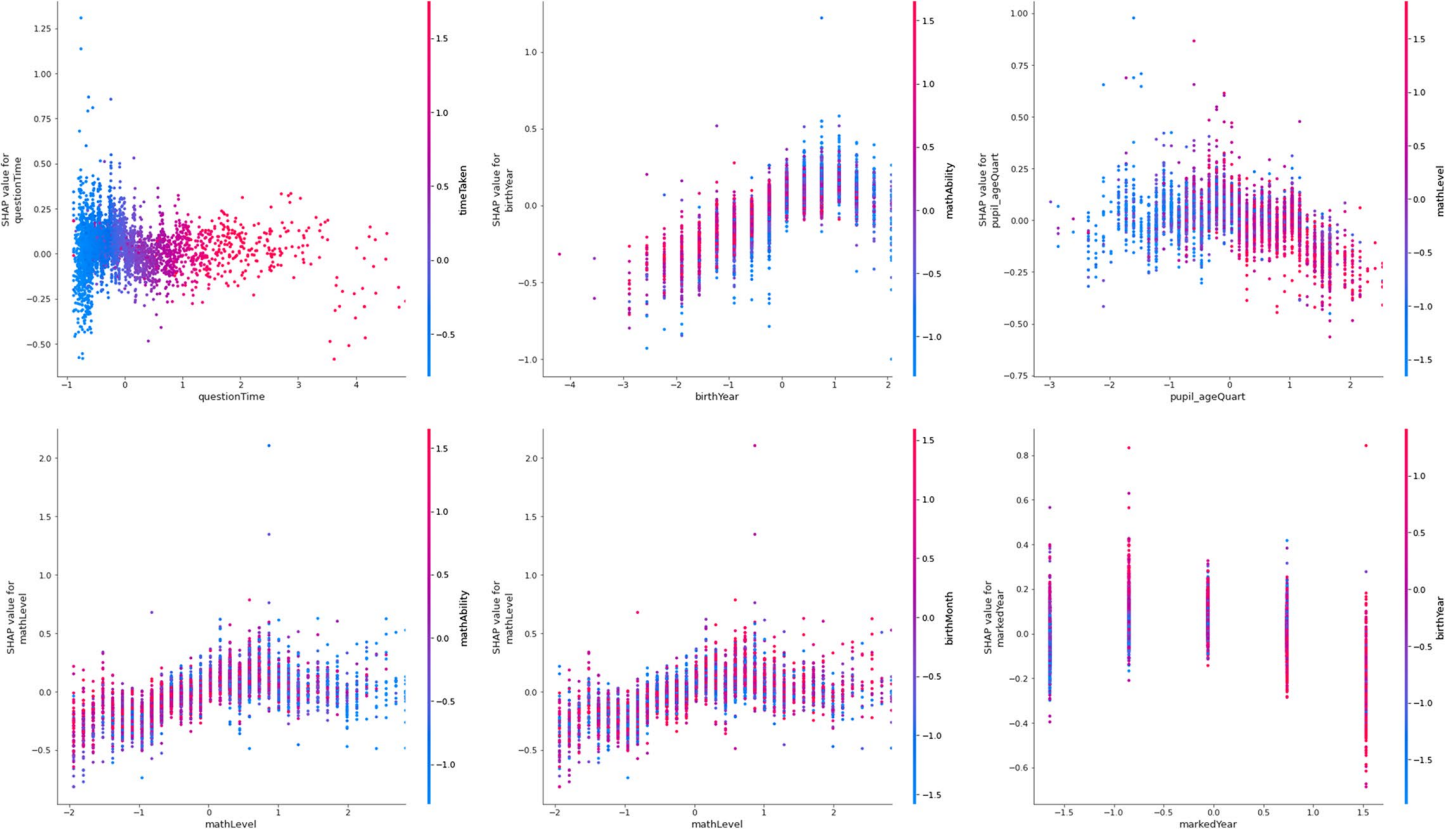
Dependence plots for the six strongest interactants to emerge from the final model when predicting access to online learning (*play_count*)

To turn to the data-led perspective, the top six interacting features are shown in Fig. 20. Of particular importance in access to online learning were the features that related to lesson difficulty (*mathLevel*) and age (*birthYear, pupil_ageQuart*) emerged from the data-led analyses. As lesson difficulty (*mathLevel*) increased with age (*pupil_ageQuart*), access to online learning decreased. Low lesson difficulty combined with high math ability to decrease access to online learning. High lesson difficulty combined with those births earlier in the year to decrease access to online learning. Other than interacting with lesson difficulty (*mathLevel*), age combined with year of access (*markedYear*) whereby older learners were less likely to access online learning after the onset of COVID-19.

The learners who completed lessons quickly (*timeTaken*) were also likely to complete questions quickly (*questionTime*): such learners were most likely to access the online learning. In contrast, those who took a long time to both complete lessons (*timeTaken*) and questions (*questionTime*) also had much lower access to online learning.

## Discussion

The present analysis deployed machine learning analysis on online learning data to investigate features relating to social justice that predict access to online learning. During theory-led model development, features relating to digital divides in access to online learning were included for model optimisation: namely, country, gender, and COVID-19. When proven necessary, data-led model development was carried out, where both feature selection and model optimisation occurred in a data-driven manner. Thus, lessons were uncovered both from domain expertise and from data-led model optimisation. By bringing both traditions together in the present data mining, rich insights have emerged to largely support the theory-led hypotheses regarding online learning, but also to supplement these with some unexpected feature patterns from the data-led perspective. In this Discussion, the narrative for the final model’s analytic outcomes will take each theory-led feature in turn (Hypotheses 1 to 3), with insights from data-led model development (Hypothesis 4) integrated into the feature-focused discussions wherever possible. In this way, the conceptual analysis of the present findings are as interpretable as possible, in accordance with the interpretable ethos of this paper.

### The role of country in access to online learning (Hypothesis 1)

Country setting was anticipated to have importance in access to online learning. This feature was supported for its potential to bring about disparities in access to online learning. In particular, Kenyan learners seemed to have the greatest disadvantage in access to online learning, as they accessed online learning notably less Kenyan and UK learners. Accordingly, it seems that infrastructural barriers are more important than English or digital literacy barriers to online learning, since it is Kenya whose infrastructure is the weakest of the three countries sampled in the present analyses (OECD, 2019; Shyu, 2022).

When country interactants were examined, a cultural effect was observed among Thai learners, where access increased with lesson difficulty. This alludes to a Southeast Asian value system which rewards achievement: family members may be permitting children to access the online learning environment increasingly, for those learners who are progressing well whereas, for those apparently progressing well through the curriculum, family may be more prohibitive. Such a pattern would be in line with the high involvement often seen in Southeast Asian families (Kurrien & Vo, 2004). The importance of family members as gatekeepers of education aligns with motivational research insights, where Thai learners enact collectivist academic motivations which is often led by others, in contrast to the individualistic online learner’s motivation who undertakes online learning for oneself rather than for others (Lim, 2004): if family members do not encourage, or if they actively discourage, children from engagement with an online learning environment, then children in Thailand are unlikely to continue accessing the resource.

The individual countries, Kenya and Thailand, were found to have particular importance in predicting access to online learning. Of the three countries analysed in the present study, these were the countries that represented LMICs and that emerged to be disadvantaged in access to online learning as individual countries. Correspondingly, LMIC status itself also emerged to have some importance in predicting access to online learning. Thus, the present study’s expectations were supported, where connectivity, electricity, English literacy, and digital literacy barriers together set learners back from accessing online learning opportunities.

The interaction analyses shed further light on disadvantages in access to online learning among LMICs. Specifically, decreasing access was observed to online learning as learners in LMICs became older. This echoes existing research documenting children’s involvement with work or labour to increase with child age in LMICs. Both in Sub-Saharan Africa and in Southeast Asia, children’s time on domestic chores (e.g., care for others in household, water or firewood fetching), market or farm work (e.g., cattle herding), or the combination of chores with farm work all increase as children’s age increases from eight, to twelve, to fifteen, to nineteen years. Correspondingly, children’s access to school and home learning both decrease with age (Keane et al., 2020). This age-related pattern is even more prominent among orphaned and abandoned children in Sub-Saharan Africa and Southeast Asia, as six-, then eight-, then eleven-year-old children increase significantly in their chances of involvement with child labour (Whetten et al., 2011).

### Gender differences in access to online learning (Hypothesis 2)

As expected, a female disadvantage was found in the present analysis of access to online learning. Thus, although a gender disparity was not found in expertise development through online learning (McIntyre, under review), the anticipated gender difference was found in this study when an equity-related outcome variable was assessed, namely educational access (Shilling, 1991), especially in LMICs (Jewitt & Ryley, 2014). This analytic outcome corresponds with existing documentation of gender disparities in access to online learning, internationally (Kashyap et al., 2020) and in LMICs (Jones et al., 2021).

Additionally, the interaction analyses suggested that, whereas boys continued to access online learning regardless of the time they took to complete a lesson, girls’ access to online learning decreased as the time they took to complete a lesson increased. It appears that girls have less time to spare for engagement with online learning. Household responsibilities may be demanding more time (Agesa & Agesa, 2019; Putnick & Bornstein, 2016) and attention (Armstrong-Carter et al., 2020) from girls than from boys. Such gendered opportunities during childhood correspond with the gendered roles displayed in adulthood among LMICs (Jeong et al., 2018), including the absence of expectation for men to engage at all with family or home responsibilities in some LMICs (Jeong et al., 2021). The experience of female disadvantage continues beyond childhood with continued impact over the life course (LeMasters et al., 2021; Qadir et al., 2011).

### The role of COVID-19 in access to online learning (Hypothesis 3)

The Pandemic was expected to have importance in rates of access to online learning: this was confirmed in the present analyses. The two features relating to COVID-19 were selected to be included in the final analytic model. Access to online learning was found simply to increase due to COVID-19, with no gesturing to issues around inequities (Fig. 9). Thus, there seemed to be a need for remote learning and this need was by and large met, during the Pandemic. In fact, online learning platforms such as the one in the present study were the most widely accessed by OECD countries during school closures (OECD, 2021).

The interaction analyses shed further light on the effect of COVID-19 on access to online learning: namely, that COVID-19 increased access to online learning when combined with lesson difficulty. COVID-19 may have brought the option of individual online learning to the foreground, especially among those who are particularly confident and competent learners of Mathematics. This corresponds with the data-led analytic outcome use of online learning increasing with each, Math ability and Math difficulty, on their own. It is not new insight that those with high self-efficacy in Mathematics tend to continue with Math learning in a virtuous circle (Bandura & Schunk, 1981). This is true regardless of geography (Bong, 2004), and whether in-person or online. In particular, student self-efficacy has been found to, together with self-monitoring, mediate the effect of student motivation on engagement with online learning (Alemayehu & Chen, 2021). To reward achievement and progress through milestones is a norm in online learning (Fig. 1). This characteristic has particular relevance to the motivation of online learners who do well in the Math online learning experience, improving their self-efficacy in particular, and motivating them to continue engagement with the online learning platform (del Rosario et al., 2020). Of course, the need for self-efficacy is balanced by the need for sufficient challenge, which is reflected in the data-led finding that learners’ access to online learning declines when learners’ ability is exceptional (Csikszentmihalyi, 2000; Patrick et al., 2006).

However, also according to the interaction analyses, access to online learning was reduced during COVID-19 among younger learners. This was somewhat surprising, as one might have expected home responsibilities to prohibit older, rather than younger, learners from access to online learning, since learner age is strongly correlated with chances of home responsibilities, and for these to diminish access to learning (Tan et al., 2022), especially in LMICs (Keane et al., 2020). Instead, COVID-19’s increase of access to online learning with learner age points to the importance of meta-cognition in online learning (Alemayehu & Chen, 2021) and the way it normally increases with age (e.g., Bryce & Whitebread, 2012), which is supported by the data-led finding that, on its own, learner age increases with access to online learning. When viewed alongside evidence that learners generally spent more time on social and leisure activities (Sevilla et al., 2020), it is conceivable that younger learners were indeed less developed in self-management capabilities and thus accessed online learning less than older counterparts.

Additionally, COVID-19 decreased access to online learning for those born at the start and end of the calendar year. *Increased* access of learners born in the start and end of the year corresponds with those who are born in the first half of the academic year. It is this group that is well-documented to have stronger academic self-efficacy and motivation than learners both in the latter half of an academic year (Givord, 2020) which reflects superior academic performance among such learners, at least during childhood (i.e., 0 to 18 years, Russell & Startup, 1986). Indeed, this was found as part of the data-led model insights. Yet, according to the interaction analyses, COVID-19 reduced access to online learning among those who otherwise enjoy stronger motivation and achievement. One explanation for this is that previously confident, high performing learners may have been so well-adjusted to learning in school that the new normal of online learning threw them off course in terms of their self-efficacy and motivation (Alemayehu & Chen, 2021; Mamolo, 2022).

### Limitations and conclusions

This study has added evidence for the large scale inequalities in access to online learning. It is important because little to no research has implemented such big data analyses to provide macro-level patterns of social justice issues in online learning. However, the contribution should be interpreted with the limitations in sight. The research, on its own, does not provide comprehensive explanations for how country, gender, and the COVID-19 pandemic brought about inequalities in access to online learning. The quantitative and aggregative nature of the present approach means that only the macro-level is seen with this single piece of research. Therefore, the present research must be viewed alongside related research on digital and sociocultural disparities in online learning.

Additionally, there was potential value in analysing multiple online learning providers’ data. One way this could have been done would have been to collate log files from multiple providers, to then bring together lessons regarding processes underlying learner performance. However, that would require find comparable experiences and metrics; it would also require that the multiple providers generate data points in the same way. Yet, there are significant between-provider idiosyncrasies in these respects. Instead, the present research focused on concerns related to social justice by examining between-country and socio-economic differences in access to online learning by a single, shared provider.

Nevertheless, this study built on existing research into online learning by augmenting it with the analysis of online learning data itself, with use of machine learning. Additionally, by employing a social justice perspective, the research findings contributed macro-level corroboration of country and gender related inequalities in access to online learning, which were exacerbated by emergency school closures during the COVID-19 Pandemic. Notably, whereas the female disadvantage was not found in previous analyses predicting the potential to develop expertise development in online learning (McIntyre, under review), the female disadvantage was found in the present analyses predicting *access* to online learning. This finding highlights how longstanding and systemic effects of world-wide disparities emerge at the macro-level of online learning outcomes, when measured as educational access. Thus, the potential to gain theoretically important insights from data science has been demonstrated, as long as theoretically rich data is obtained and integrated into model development with data obtained that represents disadvantaged populations (inc. regional granularity): this is possible when digital learning platforms from which such data is derived has been contextualised via co-development alongside local stakeholders. Moreover, theory (domain expertise) and data work must together in model development and interpretation with support from interpretable metrics and visualisations.

## Figures and Tables

**Fig. 21:**
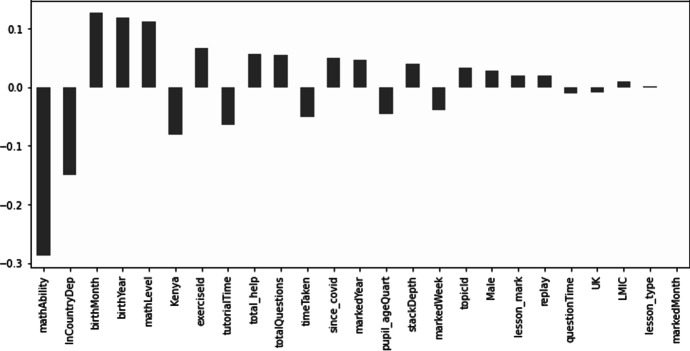
Coefficients emerging from the regularised regression model with Elastic Net penalty. Transformed data are represented here

## Data Availability

The data analysed during the current study are not publicly available due to data ownership by Math Whizz but availability can be discussed with the corresponding author upon reasonable request.

## Appendix

### Model development

#### Baseline model performance

Before development of the machine learning learning model began, a baseline model was run. This would be the model which subsequent models would increasingly outperform at each stage of model modification. For this model, the *mean* of the target variable (*play_count*) was used to train and predict the target variable. After this, the development of the analytic model was iterative. In training, this model obtained RMSE = 1.01 and Adj R^2^ = -1.37. In testing, the model obtained RMSE = 0.94 and Adj R^2^ = -1.18.

#### Phase 1: Theory-led model development

At *Stage 1*, a linear regression model was run*.* In training, this model obtained RMSE = 0.98 and Adj R^2^ = -17.65. In testing, the model obtained RMSE = 0.91 and Adj R^2^ = -14.82. Negative fit performances suggested irrelevance of the selected features. In fact, the performance (i.e., fit) was even worse than the baseline model.

At *Stage 2*, an Elastic Net model with the same features and target variable was run with cross-validation in order to identify the appropriate regularisation technique: the optimal L1 ratio = 0.50, which was between zero and one. This meant that an Elastic Net model was required, to combine the Ridge regression penalty with the Lasso penalty. With the parameters recommended from cross-validation, the Elastic Net model yielded a training RMSE = 0.98, training Adj R^2^ = -18.38; the test RMSE = 0.91 and the test Adj R^2^ = -15.43. So, performance remained inadequate.

At *Stage 3*, XGBoost^4^ was applied to further strengthen performance and to address the inadequate fit thus far. The default model (*XGRegressor()*) yielded a training RMSE = 654.29 and training Adj R^2^ = -13.45; the test RMSE = 601.58 and the test Adj R^2^ = -13.49. Thus, although performance now exceeded that of the baseline model, it remained suboptimal.

Therefore, *Stage 4* commenced the hyperparameter tuning of the XGBoost regression model. In particular, the booster hyperparameter was given attention in the first instance. In contrast to the booster = gbtree setting (the default setting and reported just above), the booster = gblinear setting resulted in very poor fit: this yielded a training RMSE = 432.43, a training Adj R2 = -29.34, a test RMSE = 417.57 and a test Adj R2 = -29.43. The overfit suggested that the booster should retain its default gbtree setting.

Next, at *Stage 5,* grid-search cross-validation was used for automated hyperparameter tuning to optimise the gradient boosting model through hyperparameter tuning. The optimised model xgb_model = XGBRegressor(learning_rate = 0.1, n_estimators = 40, max_depth = 3, min_child_weight = 6, gamma = 0, subsample = 0.8, colsample_bytree = 0.6, reg_lambda = 1, reg_alpha = 1) yielded a training RMSE = 654.74 and training Adj R2 = -20.92; the test RMSE = 604.93 and the test Adj R2 = -17.47. Again, the model remained suboptimal, especially when inspecting the model fit onto the training data.

Finally, at *Stage 6*, manual hyperparameter tuning (i.e., without automation support such as cross-validation) was conducted: this was performed on the default rather than the tuned model due to the superior performance of the former over the latter. The final manual hyperparameter tuning resulting in the final theory-led model: XGBRegressor(n_estimators = 1000) and yielded a training RMSE = 654.29 and training Adj R^2^ = -13.45; the test RMSE = 601.58 and the test Adj R^2^ = -13.49. The theory-led model was resistant to improvement, with a persistent negative performance (i.e., training Adj R2) suggesting that a theory-only was insufficient. A data-led approach to model development was necessary.

#### Phase 2: Data-led model development

At *Stage 1* of the data-led model development, a linear regression model was run with all 25 features. This model resulted in the training RMSE = 0.92 and Adj R^2^ = -26.56; the test RMSE = 0.88 and the test Adj R^2^ = -26.06.

At *Stage 2*, data-led feature selection began through regularised regression with cross-validation. Specifically, the Elastic Net regression penalty was applied during cross-validation to identify whether the Lasso penalty, Ridge regression penalty, or a combination of those via Elastic Net. With these 25 potential features in the model, Elastic Net cross-validation revealed a L1 ratio of 0.50, thereby indicating that the Elastic Net penalty would be optimal. The model underwent cross-validation again, now with the parameters suggested through cross-validation. From that, features emerging with coefficient values that were above the absolute zero were brought into the next model (see Table 1 and Fig. 21 below): these were *mathAbility, InCountryDep, birthMonth, birthYear, mathLevel,Kenya, exerciseId, tutorialTime, total_help, totalQuestions,timeTaken, since_covid, markedYear, pupil_ageQuart,stackDepth, markedWeek, topicId, Male, lesson_mark, replay, questionTime, UK, LMIC, lesson_type.* With only these features, and with the tuned model parameters, the fit improved but not sufficiently: training RMSE = 0.92, Adj R2 = -25.57, test RMSE = 0.88, test Adj R2 = -25.06. Therefore, boosting was performed as the next stage in model development.^5^

At *Stage 3*, XGBoost was applied to a regression model identified during Stage 2 as part of the data-led approach to feature selection: *mathAbility, InCountryDep, birthMonth, birthYear, mathLevel,Kenya, exerciseId, tutorialTime, total_help, totalQuestions,timeTaken, since_covid, markedYear, pupil_ageQuart,stackDepth, markedWeek, topicId, Male, lesson_mark, replay, questionTime, UK, LMIC, lesson_type*. The default model (*XGRegressor()*) yielded a training RMSE = 0.28 and training Adj R^2^ = 0.90; the test RMSE = 0.89 and the test Adj R^2^ = 0.92. With XGBoost, performance dramatically improved. Further tuning was explored, to see if the fit would improve even further.

At *Stage 4*, one hyperparameter in the XGBoost model underwent tuning, namely the *booster* hyperparameter. In contrast to the above parameter setting which is default (*booter* = *gbtree*), the setting *booster* = *gblinear* used linear functions and yielded a training RMSE = 0.92 and training Adj R^2^ = -3.60; the test RMSE = 0.88 and the test Adj R^2^ = -3.67. The overfit suggested that the booster should retain its default *gbtree* setting.

At *Stage 5*, the XGBoost model underwent systematic, automated hyperparameter tuning. The *learning rate* (= 0.1) was chosen as the parameter to start with that would be held constant: the optimal number of values for this learning rate (*n_estimators*) was tested. *Learning rate* and *n_estimators* were then held constant until the end while (1) *max_depth* and *min_child_weight*, (2) *gamma*, (3) *subsample* and *colsample_bytree*, and the (5) regularisation parameters were tuned. Finally, (6) the *n_estimators* and (7) the *learning rate* themselves were tuned. Automated hyperparameter tuning was conducted via the *grid search CV* technique, which performs an exhaustive search over every specified hyperparameter value was conducted via the grid search cv method. This tuning resulted in the model, xgb_model = XGBRegressor(learning_rate = 0.1, max_depth = 4, min_child_weight = 8, gamma = 0.2, colsample_bytree = 0.65, subsample = 0.65, reg_lambda = 0.05, reg_alpha = 0). This yielded the model fit with the training RMSE = 0.74, training Adj R2 = -0.75, test RMSE = 0.83, test Adj R2 = -0.77. Since this automated hyperparameter tuning did little to improve the model performance, the default algorithm was brought forward for tuning.

At *Stage 6*, the default XGBoost algorithm was tuned manually and resulted in this model: XGBRegressor(learning_rate = 0.60). Performance strengthened to yield a training RMSE = 0.15 and training Adj R^2^ = 0.98; test RMSE = 0.96 and test Adj R^2^ = 1.00. This is the model to be reported in the Results section. As before and throughout model development, the outcome variable was *play_count.*

## Footnotes

1 The English Proficiency Index (EPI) for Thailand is classified as ‘Very Low’ (EPI = 419) by Education First (2021), and can be compared with that of Kenya (EPI = 587) where EPI is classified as ‘High’.

2 Note that the cited study precedes the present analysis, as the present work interrogates the first paper’s findings by adopting a social justice framework to the theory-led model development for analysis of online learning.

3 In support of the country income status allocations in this paper, the gross domestic product (GDP) indices are provided for year 2021: Kenya (LMIC) = 1.10 E + 11; Thailand (LMIC) = 5.06 E + 11; UK (HIC) = 3.19 E + 12 (World Bank, 2021).
